# Interventions to Address Disparities in Perinatal Outcomes by Ethnicity: A Systematic Review

**DOI:** 10.1111/1471-0528.70013

**Published:** 2025-09-23

**Authors:** Sara Sorrenti, Smriti Prasad, Nouran Elbarbary, Fathima Fidha, Laura A. Magee, Peter von Dadelszen, Sergio A. Silverio, John Allotey, Shakila Thangaratinam, Asma Khalil

**Affiliations:** ^1^ Fetal Medicine Unit Liverpool Women's Hospital Liverpool UK; ^2^ Department of Maternal and Child Health and Urological Sciences Sapienza University of Rome Rome Italy; ^3^ Fetal Medicine Unit St George's Hospital London UK; ^4^ Department of Women & Children's Health, School of Life Course & Population Sciences King's College London London UK; ^5^ Institute of Women and Children's Health King's Health Partners London UK; ^6^ Department of Psychology, Institute of Population Health University of Liverpool Liverpool UK; ^7^ Institute of Life Course and Medical Sciences University of Liverpool Liverpool UK; ^8^ Liverpool Women's NHS Foundation Trust Liverpool UK; ^9^ NIHR Applied Research Collaboration Northwest Coast Liverpool UK; ^10^ Vascular Biology Research Centre, Molecular and Clinical Sciences Research Institute City St George's University of London London UK; ^11^ Royal College of Obstetricians and Gynaecologists London UK

**Keywords:** equity, ethnic minorities women, ethnic minority, health disparities, pregnancy in women of ethnic minorities

## Abstract

**Background:**

Ethnic minority women face disproportionately higher risks of adverse perinatal outcomes, exacerbated by socio‐economic and systemic barriers.

**Objectives:**

This systematic review evaluates the effectiveness of interventions designed to improve perinatal outcomes in these populations.

**Search Strategy:**

We conducted a systematic review according to a pre‐designed protocol (PROSPERO CRD42024516616). MEDLINE, EMBASE and Cochrane Databases were searched up to February 2024 using relevant Medical Subject Headings (MeSH) terms and keywords.

**Selection Criteria:**

We included studies involving interventions targeting pregnant women from ethnic minority groups. Outcome measures included maternal and perinatal outcomes, as well as qualitative assessments, when available.

**Data Collection:**

Two reviewers independently performed data extraction and quality assessment, resolving discrepancies by consensus.

**Main Results:**

Studies included (*n* = 36) were from the United Kingdom (*n* = 9), United States of America (*n* = 9), Australia (*n* = 12), Canada (*n* = 1), Denmark (*n* = 2), Sweden (*n* = 3), involving women (*n* = 72 527) of varied ethnicity: Asian (*n* = 16 274, 22.4%), Black (*n* = 11 458, 15.8%), Hispanic (*n* = 612, 0.8%), First Nations/Aboriginal (*n* = 19 406, 29.1%), Mixed (*n* = 873, 1.2%), ‘Other’ (as defined in the included studies) (*n* = 3354, 4.6%), and women belonging to an unspecified ethnic minority group (*n* = 15 232, 21%), and a group of Russian, Arabic, Tigrinya, Polish and Somali women in a foreign country (82 women; 0.1%). Interventions broadly included four categories: clinical management interventions, educational programmes, treatments, and models of care. Clinical management interventions like increased foetal surveillance after 39 weeks and implemented screening for preeclampsia showed positive results, with a 64% reduction in stillbirth rates among South Asian (aOR 0.36, 95% CI 0.13–0.90, *p* = 0.047) with the former intervention, and a decrease in perinatal deaths with the latter intervention. Educational initiatives demonstrated diverse results, with those directed to the families showing significant improvement in satisfaction and active participation in prenatal care; however, no significant improvements were noted after the implementation of initiatives devoted to healthcare providers. Specific treatments, such as low‐dose aspirin, have yielded various outcomes, with some studies reporting a reduction in preterm birth rates. Models of care, including midwifery continuity of care, nutrition implementation initiatives, home visits and language support services, showed promising results in improving maternal satisfaction and obstetric outcomes.

**Conclusions:**

This systematic review summarises the interventions to improve outcomes for these families among ethnic minority women and emphasises the lack of focused attention on improving outcomes in these groups, highlighted by the limited studies and the diverse interventions and outcomes reported. While educational and social support programmes within the model of care show promise, large‐scale and high‐quality studies are needed.

## 1 Background

Ethnic disparities in healthcare outcomes, particularly in maternal and perinatal contexts, have been a persistent and significant public health concern. This has been repeatedly highlighted in the United Kingdom (UK) Confidential Enquiry into Maternal Death reports [1, 2]. The 2018 report served as a catalyst for the formation of focused campaign groups, such as Fivexmore [3, 4], but in 2023, Black women in the UK were still 2.8 times more likely to die during pregnancy and childbirth compared to White women, as well as Asian women (1.67 times higher mortality than White women) [1]. Ethnic disparities in pregnancy outcomes are not confined to the UK [5, 6, 7], and are observed in other high‐income countries, including the United States of America (USA), Australia and member states of the European Union. In the USA, the Centre for Disease Control reports have highlighted that the cause‐specific pregnancy‐related mortality ratio (PRMR) was higher for Non‐Hispanic Black (40.8) and Non‐Hispanic American Indian/Alaska Native (29.7) women, compared to the overall PRMR of 16.7 pregnancy‐related deaths per 100 000 births [8]. Population‐based data from Australia, Canada and countries of the European Union showed similar trends [9, 10, 11, 12].

Women belonging to ethnic minority groups often face challenges and barriers in accessing quality healthcare, which can adversely affect pregnancy outcomes [13, 14, 15]. These challenges include socio‐economic disadvantages, literacy barriers [16, 17], cultural differences [18] and structural and systemic biases within healthcare systems [19, 20]. A unique situation is represented by the First Nation women in Australia, for example, as a result of colonisation, where they still represent a minority in the countries they inhabit. In recent years, these inequities have been brought to the attention of the scientific community, especially after the challenges highlighted by the COVID‐19 pandemic [21, 22, 23, 24, 25, 26, 27].

Targeted interventions are required to improve inequities in maternal and perinatal health outcomes for ethnic minority group women [28].

In this systematic review, we provide a comprehensive overview of the types of interventions that have been implemented to improve pregnancy outcomes for ethnic minority women.

## 2 Methods

### 2.1 Protocol, Information Sources and Literature Search

This systematic review was conducted according to a pre‐designed protocol and registered in the PROSPERO database (registration number CRD42024516616). PRISMA guidelines were followed [29] (Supporting Information S1).

Articles of interest were identified by electronic search of MEDLINE, EMBASE, Cochrane Database of Systematic Reviews and Cochrane Central Register of Controlled Trials, which were searched electronically from inception up to 2 February 2024, utilising combinations of the relevant medical subject heading (MeSH) terms and free‐text terms (Appendix S1). An update of the search was performed in July 2024, utilising additional reference lists to search for relevant studies.

The search and selection criteria were restricted to the English language.

### 2.2 Outcomes Measures, Study Selection and Data Collection

All studies involving any intervention, policy, or secondary analyses of interventions designed to improve perinatal outcomes in ethnic minority pregnant women were included. Ethnic minorities were considered a particular group of people sharing culture, tradition, language and origins, living in a country where most of the population belongs to a different ethnic group (*Definition of ethnic minority from the Cambridge Advanced Learner's Dictionary & Thesaurus Cambridge University Press*). Inclusion criteria referred to the following PICO model: the study population included pregnant women belonging to a specific ethnic minority; for the purpose of this review, women with broader socioeconomic disadvantages were excluded as we believed that, despite the degree of overlap between these populations, the latter face different challenges that are not necessarily related to the ethnic background. As an intervention, all initiatives aimed at improving prenatal care in the population group (i.e., models of care, training initiatives and specific treatments, …) were included. The comparison group included, when available, women of ethnic minorities who did not receive the intervention; the main outcomes included pregnancy and perinatal outcomes (i.e., preterm birth, stillbirth, Caesarean section, preeclampsia, neonatal death and Apgar score, …).

Three authors (S.P., S.S., N.A.) independently reviewed the abstracts and full texts. Disagreement was resolved by consensus.

### 2.3 Quality Assessment of Included Studies

Quality assessment of included studies was performed using the Newcastle‐Ottawa Scale (NOS) for cohort studies [30] and the TRACT assessment tool for randomised controlled trials [31].

According to NOS, each study is evaluated based on the selection of the study groups, the comparability of the groups, and the ascertainment of the outcome of interest [26].

TRACT assessment (Checklist to assess Trustworthiness in Randomised Controlled Trials) was used to evaluate RCTs included in the present meta‐analysis. The TRACT checklist analyses seven domains for signs of compromised research integrity: governance, author group, plausibility, timeline, drop‐out rates, balance of baseline characteristics and study outcomes and effect sizes [27].

Two investigators (S.S., N.A.E.) assessed the quality of the included studies.

### 2.4 Data Extraction and Statistical Analysis

Two authors (S.S. and N.A.E.) extracted data for each study independently. A narrative approach was adopted to present data in the results section, according to the type of intervention. Given the high heterogeneity among the interventions investigated in the included studies, and the different populations included, we were not able to perform a meta‐analysis of similar studies with an acceptable degree of concordance. On the other hand, with the narrative approach, we were able to conduct a comprehensive review of different interventions among different study groups reported in the current literature. Results were presented according to the type of intervention, in four broad groups: clinical management interventions, education, treatment and models of care. We chose this approach to simplify the reading of the results and focus on the types of interventions that showed promise. Analysis was restricted to descriptive statistics and performed using Microsoft Excel 2019.

## 3 Results

### 3.1 Study Selection and Characteristics

**FIGURE 1:**
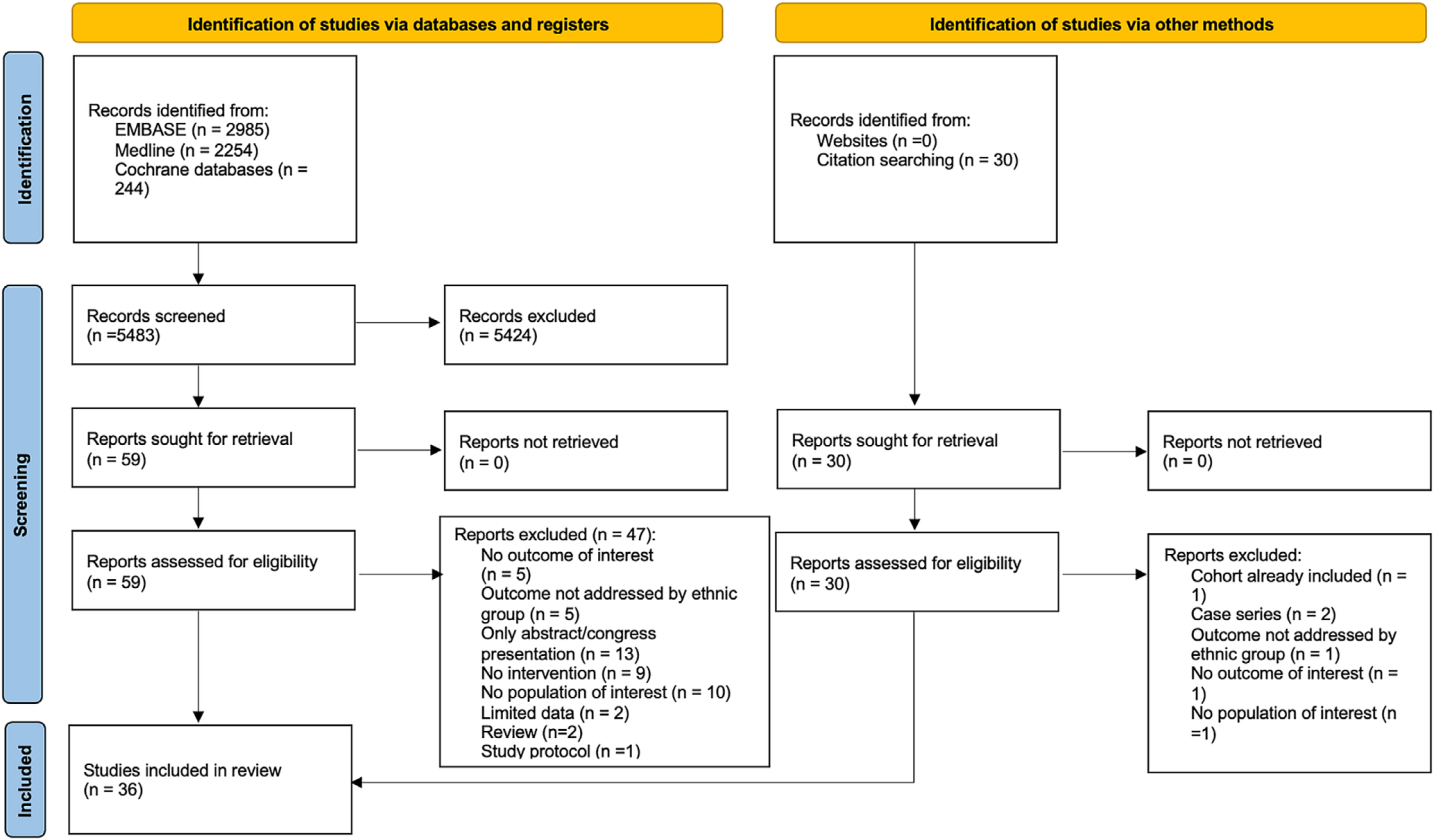
PRISMA 2020 flowchart.

The search identified 5483 articles after removing duplicates, of which 62 articles were selected for full‐text review (Figure 1). After review, 36 studies were eligible for inclusion [32, 33, 34, 35, 36, 37, 38, 39, 40, 41, 42, 43, 44, 45, 46, 47, 48, 49, 50, 51, 52, 53, 54, 55, 56, 57, 58, 59, 60, 61, 62, 63, 64, 65, 66, 67]. A list of excluded studies is available in Table S1.

Characteristics of the included studies are displayed in Table 1. Twenty‐five studies were observational cohort studies, either retrospective (*n* = 17) [32, 33, 38, 39, 45, 46, 47, 49, 50, 51, 52, 54, 55, 58, 60, 61, 62], or prospective (*n* = 8) [37, 40, 48, 53, 56, 57, 63, 64], other studies were randomised controlled trials (*n* = 4) [42, 59, 65, 66], secondary analyses of RCTs (*n* = 5) [34, 35, 36, 41, 43], a secondary analysis of a case–control study (*n* = 1) [44] and an interventional‐controlled study [67]. The most recent study was published in 2023 [32], and the least recent in 1981 [65]. Studies were primarily from Australia (*n* = 12) [32, 37, 40, 46, 47, 49, 51, 56, 57, 58, 60, 61], UK (*n* = 9) [33, 38, 39, 45, 50, 62, 63, 64, 65], USA (*n* = 9) [32, 36, 37, 40, 41, 43, 44, 46, 47, 48, 49, 51, 52, 53, 55, 56, 57, 58, 59, 60, 61, 66, 67].

**TABLE 1:** Characteristics of the included studies.

Authors	Year	Study period	Country	Study design	Population (ethnic minority)	Study group	Control group	Intervention
Davies‐Tuck et al. [32]	2023	2016–2020	Australia	Retrospective cohort study	South Asian	8532 (after clinical change)	3506 (before clinical change)	Foetal surveillance from 39 weeks (clinical practice change)
Ahrne et al. [67]	2023	2016–2019	Sweden	Interventional‐control study	Somali	64 Somali	81 Somali	Group antenatal care vs. standard antenatal care
Muller et al. [33]	2023	2018–2021	UK	Retrospective cohort study	South Asian, Black, Mixed, Others (except white)	3590 South Asian; 1253 Black; 788 Mixed; 1526 others	36 421 South Asian; 15 089 Black; 7139 Mixed; 18 398 others	Induction of labour at 39 weeks in low‐risk women versus expectant management
Rasmussen et al. [34]	2023	2018–2019	Denmark	Nationwide register‐based analysis of MAMAACT trial (RCT)	Immigrants from low and middle‐income countries (Middle East, Sub‐Saharan Africa, South Asia and Eastern Europe)	10 775 pre‐implementation; 4060 post‐implementation	7802 pre‐implementation; 2754 post‐implementation	Midwives' education (MAMAACT intervention) in intercultural communication and cultural competence
Rasmussen et al. [35]	2023	2018–2019	Denmark	Cross‐sectional questionnaire of women in the MAMAACT trial (RCT)	Non‐Western immigrants	180 pre‐implementation; 217 post‐implementation	116 pre‐implementation; 157 post‐implementation	Midwives' education (MAMAACT intervention) in intercultural communication and cultural competence
Kane et al. [36]	2023	(RCT 1989–1991)	USA	Secondary analysis of RCT	Hispanic, Black, Others	400 Hispanic, 641 Black, 9 Other	419 Hispanic; 624 Black; 10 Other	Low dose aspirin in the prevention of preterm birth
Berman et al. [37]	2023	2009–2019	Australia	Prospective population‐based cohort study	Aboriginal	19 406 Aboriginal	Aboriginal before the WA Initiative	Western Australia (WA) Initiative: new guidelines for reducing rate of preterm birth in singletons
Liu et al. [38]	2022	2016–2020	UK	Retrospective cohort study	Black, Asian, Mixed/Others	1440 Black, 2330 Asian, 579 Mixed/Others	941 Black, 1518 Asian, 307 Mixed/Other had standard NICE screening	FMF screening for preeclampsia
Schytt et al. [66]	2022	2018–2020	Sweden	RCT	Polish, Somali, Russian, Arabic, Tigrinya	82 of all these ethnic groups	68 of all these ethnic groups	Community‐based bilingual doula (CBD) support in labour and post‐partum
Hadebe et al. [39]	2021	2018–2020	UK	Retrospective cohort study	BAME (Black, Asian and Minority Ethnic community) living in aeras of social depravation	66 Black, 6 Asian, 3 Chinese, 14 mixed, 21 Other	79 Black, 11 Asian, 2 Chinese, 16 Mixed, 17 Other	Targeted caseload midwifery
Kildea et al. [40]	2021	2013–2019	Australia	Prospective intervention trial	First Nations Australians	766 mothers of First Nations babies (88% First Nations women; others: partners of First Nations fathers)	656 mothers of First Nations babies (61% First Nations women; others: partners of First Nations fathers)	Birthing in Our Community (BiOC) service
Andrikopoulou et al. [41]	2021	RCT (1996–2000)	USA	Secondary analysis of a RCT	Black, Asian, Other/mixed	311 Black, 48 Asia, 141 Others	316 Black, 35 Asian, 147 Others	Betamethasone administration in late preterm
Akselsson et al. [42]	2020	2016–2018	Sweden	Cluster randomised trial	Women born in Somalia	169 Somali born	454 Somali born (routine care)	Intervention to promote active role of pregnant women to daily self‐monitoring of foetal movements (Mindfetalness)
Tolcher et al. [43]	2020	1991–1995	USA	Secondary analysis of RCT	Hispanic, non‐Hispanic Black, Other	Low‐risk group: 23 Hispanic, 42 Black, 1 Other; High‐risk group: 21 Hispanic, 136 Black, 1 Other	Low‐risk group: 26 Hispanic, 50 Black, 1 Other; High‐risk group: 25 Hispanic, 137 Black, 0 Other (placebo)	Low dose aspirin for preeclampsia prevention
Angley et al. [44]	2018	2006–2008	USA	Secondary analysis of a case–control study	Hispanic, non‐Hispanic Black	—	—	Special Supplemental Nutrition Program for Women, Infants and Children (WIC)
Homer et al. [45]	2017	1997–2009	UK	Retrospective study	Black, Asian and Minority Ethnic (BAME) communities	847 Black African, 245 Black Caribbean, 147 Black British, 217 Asian, 71 Mixed, 93 Other	No control group	Albany Midwifery Practice
Middleton et al. [46]	2017	2010–2012	Australia	Retrospective study	Aboriginal	486	1452	Aboriginal Family Birthing Program (AFBP)
Reeve et al. [47]	2016	2007–2010	Australia	Retrospective study	Aboriginal and Torres Strait Islander (Indigenous)	121	92	Implementation of a midwifery‐led interdisciplinary model of antenatal outreach care
Tandon et al. [48]	2013	2008–2009	USA	Prospective study	Hispanic	144	70	Centering Pregnancy group prenatal care model
Kildea et al. [49]	2012	2004–2009	Australia	Retrospective study	Aboriginal and Torres Strait Islander (Indigenous)	367 (included women partnered to Indigenous men)	414 (included women partnered to Indigenous men)	Murri Antenatal Clinic
Murphy et al. [50]	2012	2000–2003	UK	Retrospective service evaluation	Aboriginal	689	—	Aboriginal Maternal and Infant Health Service
Wong et al. [51]	2011	2004–2008	Australia	Descriptive retrospective study	Aboriginal	130	471	Aboriginal Midwifery Access Program (AMAP)
Khanani et al. [52]	2010	2005–2008	USA	Retrospective cohort study	African‐American; Other	5731 African‐American, 980 Other	3335 African‐American, 1176 Other	Special Supplemental Nutrition Program for Women, Infants and Children (WIC)
Robertson et al. [53]	2009	Not specified	USA	Prospective study	Hispanic	24	25	Centering Pregnancy Model (CPM)
Simonet et al. [54]	2009	1989–2000	Canada	Geocoding‐based retrospective birth cohort study	Inuit	Inuit residents in the Hudson Bay (midwives were the primary birthing attendants)	Ungava Bay communities (physicians were the primary birthing attendant)	Midwife‐led maternity care
Wells et al. [55]	2008	2003–2005	USA	Retrospective cohort study	African‐American	48	61	Antepartum nurse case management home visitation
Panaretto et al. [56]	2007	2000–2005	Australia	Prospective study	Indigenous	781	84	Mums and Babies program
Panaretto et al. [57]	2005	2000–2003	Australia	Prospective study	Indigenous	456	540	Mums and Babies program
D'Espaignet et al. [58]	2003	1988–2001	Australia	Retrospective study	Aboriginal	Group 1 (829) and Group 2 (322) in different communities	Group 1 (3070) and Group 2 (3511)	Strong Women, Strong Babies, Strong Culture Program (SWSBSC)
Klerman et al. [59]	2001	1994–1996	USA	Randomised trial	African‐American	318	301	Augmented prenatal care
Mackerras et al. [60]	2001	1990–1991 and 1994–1996	Australia	Retrospective study	Aboriginal	246 post‐implementation	228 pre‐implementation	Strong Women, Strong Babies, Strong Culture Program (SWSBSC)
Smith et al. [61]	2000	1991–1996 and 1996–1997	Australia	Retrospective study	Aboriginal	43 post‐implementation	204 pre‐implementation	Two interventions: Implementation of Strong Women, Strong Babies, Strong Culture Program (SWSBSC) and nutritional support intervention for pregnant women and mothers of young children
Parsons et al. [62]	1992	1984–1986	UK	Retrospective study	Asian, Turkish	1000	992	Multi‐Ethnic Women's Health Project (MEWHP)
Mason et al. [63]	1990	1985–1987	UK	Prospective study	Asian	457	—	Asian Mother and Baby Campaign
McEnery et al. [64]	1986	1980–1981	UK	Prospective study	Asian	35	34	Antenatal education
Maxwell et al. [65]	1981	—	UK	Randomised trial	Asian	59	67	Vitamin D supplementation (1000 IU/day)

Overall, 72 527 women from various ethnic groups participated in the included studies (Table 1). Most often, participants were Asian (16 274 women, 22.4%; 10 studies) [32, 33, 38, 39, 41, 45, 62, 63, 64, 65], Black (11 458 women, 15.8%; 15 studies) [33, 36, 38, 39, 41, 42, 43, 44, 45, 52, 55, 59, 67], or First Nations/Aboriginal (24 642 women, 34%; 12 studies) [37, 40, 46, 47, 49, 50, 51, 56, 57, 58, 60, 61]. Other participants were Hispanic (612 women, 0.8%; 5 studies) [36, 43, 44, 48, 53], Mixed origin (873 women, 1.2%; 3 studies) [33, 38, 41], ‘Other’ (as indicated by the authors of the included studies) (3354 women, 4.6%; 5 studies) [33, 36, 38, 41, 43], and not specified (only defined as ‘Immigrants’ by authors of the included studies) (15 232 women, 21%; 2 studies) [34, 35]. One study reported a group of Russian, Arabic, Tigrinya, Polish, and Somali women in a foreign country (82 women; 0.1%; 1 study) [66].

Investigated interventions varied and included: (i) diagnostic pathways or management of pregnancy (*n* = 7 studies) [32, 33, 37, 38, 39, 47, 54]; (ii) educational interventions offered to health operators (doctors, nurses, midwives, …) or women (*n* = 6 studies) [34, 35, 42, 46, 48, 64], (iii) a specific treatment during pregnancy (*n* = 4 studies) [36, 41, 43, 65]; or (iv) different models of care offered to ethnic minority women (*n* = 19 studies) [40, 44, 45, 49, 50, 51, 52, 53, 55, 56, 57, 58, 59, 60, 61, 62, 63, 66, 67] (Figure 3). Interventions were broadly divided into these categories following a preliminary review of the available literature and pragmatic considerations to create coherent categories to facilitate a more structured analysis.

Quality assessment of included studies is available in Table S2. The quality of the included studies was overall good. Low comparability was attributed to 10 of the included studies, mainly due to the lack of analysis of the results or the inclusion of historical cohorts as control groups. In fact, the low comparability of the studies was the main limitation to the performance of a high‐quality meta‐analysis. The TRACT assessment showed no concerns for all the randomised trials included in the present review, except for one showing some concerns in the governance and plausibility of intervention [31].

### 3.2 Synthesis of the Results

Tables S3–S5 presents detailed information about the results. Table S5 illustrates the varied effectiveness of targeted interventions on perinatal outcomes across different ethnic minority groups.

#### 3.2.1 Clinical Management Interventions

Seven studies reported outcomes in pregnant women after a change in clinical practice as an intervention. These included enhanced foetal surveillance with cardiotocography and assessment of amniotic fluid volume [32], screening and prevention of preterm birth [37], screening for preeclampsia [38], induction of labour at 39 weeks [33], and midwife‐led maternity care among ethnic minority women [39, 54]. These interventions showed mixed results: the former intervention determined a reduction in stillbirth (aOR 0.36, 95% CI 0.13–0.90) and fewer early neonatal deaths (3.1/1000 vs. 1.3/1000 after intervention) [32]; the implementation of midwifery‐led care in one study was associated with higher rates of first‐trimester first accesses in pregnancy (58% vs. 40%, *p* = 0.01) and a higher number of ultrasound scans in pregnancy (94% vs. 59%, *p* < 0.001) [47], whereas other authors found that this intervention was associated with a significant reduction in the rate of Caesarean sections (27.8% vs. 43.1%; RR 0.68, 95% CI 0.47–0.99) [39]. On the contrary, the other interventions showed no impact on the considered outcomes [37, 54] among ethnic minority groups, except for the screening for preeclampsia, which showed a significant decrease in the perinatal death rate only among Asian women [38].

#### 3.2.2 Education

Educational interventions were the subject of six studies. Three of these evaluated the education of healthcare providers [34, 35], whereas the other three investigated the role of education in pregnant women [42, 48, 64].

Two studies analysed the results of the RCT of the MAMAACT intervention, which consisted of a training programme for midwives on intercultural communication and cultural knowledge, and an educational campaign for the identification of warning signs in pregnancy directed to women, in different languages [34, 35]. Authors found a significant reduction in the rate of neonates born with arterial pH < 7 (aOR 0.27, 95% CI 0.09–0.81) and a significant increase in the rate of NICU admission (aOR 1.36, 95% CI 1.05–1.76) [34]. The MAMAACT intervention was also studied as a determinant of active engagement of the ethnic minority women with the healthcare professionals and increased awareness of the use of healthcare informatic systems and the recognition of alerting symptoms [35]. No significant improvements were observed in any of the mentioned outcomes [35]. Similarly, no improvements were noted in the other educational intervention directed to healthcare providers (Aboriginal Family Birthing Programme [AFBP]) [46].

On the other hand, the educational programs for pregnant women, consisting of lessons to the Asian community, a program called ‘Mindfetalness’ aiming to improve maternal awareness of foetal movements, showed promising results: higher rates of continuous breastfeeding (48% vs. 31%) and vaccination uptake for infants (100% vs. 72%) [64] lower rates of PTB (0.6% vs. 3.1%, aRR 0.15, 95% CI 0.01–0.75) [42], and significant improvement in satisfaction, active participation in prenatal care, satisfaction with time spent talking with the provider, and ability to speak with their prenatal care provider in the preferred language [48].

#### 3.2.3 Treatment

Specific treatments administered to ethnic minority pregnant women were investigated in four studies. Two of them regarded the role of LDA [36, 43]. The first analysed the effect of this intervention in preventing preterm birth [36], and demonstrated that, at baseline, Black race was independently associated with increased risk of PTB < 37 and < 34 weeks compared to the White race and that the adjustment for treatment eliminated these differences between ethnic groups [36]. Therefore, the authors speculated that LDA may play a positive role in reducing the risk of PTB in these women [36]. The second study aimed to assess the role of LDA in preventing pre‐eclampsia [43]: no significant effects were observed in the rate of pre‐eclampsia after administration of LDA (60 mg) in low‐risk or high‐risk Hispanic and non‐Hispanic Black women (RR 0.740, 95% CI 0.550–1.010 in low‐risk and RR 0.91, 95% CI 0.77–1.06 in high‐risk women) [43].

The other two studies investigated the role of Betamethasone in late PTB and showed that race was not predictive of the primary outcome (need for respiratory support within 72 h after birth) in the placebo or study group [41]; and the supplementation of Vitamin D (1000 IU/day), which was associated with greater weight gain in the third trimester (63.3 ± 20.7 g/day vs. 46.4 ± 29.5, *p* < 0.001) and higher concentrations of thyroid binding prealbumin (18.5 ± 3.5 vs. 14.8 ± 3.4, *p* < 0.01) and retinol binding protein (4.3 ± 1.6 vs. 3.7 ± 1.0, *p* < 0.05), but no significant differences in the rate of low birthweight infants (11.8% vs. 22.3%) [65].

#### 3.2.4 Models of Care

Nineteen studies examined the impact of different models of care and support initiatives to reduce inequality in access to healthcare among ethnic minorities. These included nutritional supplementation programs [44], that were shown to have a positive impact on the reduction of stillbirth in the non‐Hispanic black group (aOR 0.34, 95% CI 0.16–0.72) [44]; other groups of African American women also experienced lower rates of PTB < 37 (13.7% vs. 20%, *p* < 0.001) and < 34 (4.0% vs. 8.1%, *p* < 0.001) weeks and lower infant death rate (9.6% vs. 21%; *p* < 0.001) after nutritional support initiatives [52, 55]. In another study, this nutritional intervention did not result in an improvement in the rate of low‐birthweight newborns [61].

Other interventions in this area included monthly visits provided by nurses (‘Black Babies SMILE’ initiative), which showed lower rates of PTB (aOR 0.31, 95% CI 0.11–0.88) [55]; interpreting services that demonstrated promising results: reduction in the rates of elective Caesarean section (2.3% vs. 5.9%), instrumental delivery (6.6% vs. 9.4%) and higher rates of spontaneous delivery (86.8% vs. 74.8%) [62].

Support services in the form of midwifery continuity of care [45], home visits, assistance with appointments, transport, birth support, postnatal follow‐up [51], support to young women by senior Aboriginal women [58] showed a positive impact on the outcomes considered: decreased perinatal mortality rates (< 2/1000 births vs. 8.8–9.8/1000 reported in previous studies) [45], slightly lower rates of Caesarean section (20% vs. 27.6%), PTB (18.8% vs. 21.6%) and low birthweight rates reported by three different studies (18.8% vs. 21% [51]; 10.9% vs. 15.3%, *p* = 0.014 [58]; and 19.8% vs. 11.3%, *p* = 0.02 [60], respectively).

Another study investigated the role of the Birthing in Our Community (BiOC) social service in improving pregnancy outcomes in Australia: First Nations Australians attending BiOC demonstrated lower rates of PTB (OR 0.42, 95% CI 0.26–0.66), low birthweight (OR 0.60, 95% CI 0.41–0.89), admission to neonatal nursery (OR 0.69, 95% CI 0.51–0.92, *p* = 0.013) and higher rates of first visit in the first trimester (OR 1.35, 95% CI 1.05–1.73) [40].

In the study conducted among Aboriginal women in Australia about the Murri Antenatal Clinic, characterised by the implementation of Aboriginal midwives and non‐Aboriginal obstetricians and social supporters, the intervention showed an increase in the number of visits in pregnancy (15.8% vs. 11% of women had 2–4 visits; and 23.6% vs. 19.7% of women had 5–7 visits, *p* = 0.007). Lower rates of NICU admissions were reported (15.9% vs. 21.8%, *p* = 0.036) [49].

The Mums and Babies program, consisting of continuity of care, daily maternal and child health clinics, integrated team approach, family orientation, screening services, transport services and intervention for risk factors, was studied as an intervention in Aboriginal pregnant women in Australia [57]. The intervention group had significantly lower rates of PTB (8.7% vs. 14.3%, *p* = 0.002) [57]. No differences in the rates of low birthweight (*p* = 0.067) or perinatal death (*p* = 0.864) were registered [57]. The same authors reported results from a larger cohort in 2007 (*n* = 781), but they used a different reference group for comparisons (historical cohort 1998–1999 vs. contemporary cohort 2000–2003, previously reported) [56]. The second study showed similar results. However, the reduction in perinatal mortality was significant (*p* = 0.014) [56].

In the UK, the implementation of the Aboriginal Maternal and Infant Health Service to improve perinatal outcomes among Aboriginal groups showed a non‐significant reduction in perinatal mortality (20.4 to 14.4 per 1000 births), along with a significant improvement in the rate of women attending their first visit in the first trimester (OR 1.2, 95% CI 1.01–1.4, *p* = 0.03) and a significant reduction in the rate of PTB (OR 0.5, 95% CI 0.4–0.8, *p* < 0.001) [50].

A similar program was implemented among African Americans in the USA, with the initiative called Augmented prenatal care, including educational support to pregnant women, smoking cessation programs, social support, prenatal care following American College of Obstetrics and Gynaecology (ACOG) guidelines, highly expert nurses in prenatal care, 2‐weekly appointments, transport services and educational materials [59]. The intervention group showed higher rates of prenatal care positive rating (94% vs. 80%, *p* = 0.002), higher number of prenatal visits (13.7 + 3.8 vs. 11.9 + 3.8, *p* = 0.001) and higher participation in prenatal courses (79% vs. 17%, *p* < 0.001) [59]. No significant differences were noted in the rates of Caesarean deliveries (*p* = 0.24), low birthweight (*p* = 0.60), PTB (*p* = 0.22), foetal growth restriction (*p* = 0.26), low Apgar score (< 7) at 1 min (*p* = 0.35) or at 5 min (*p* = 0.52) and mean gestational age at delivery (*p* = 0.19) [59].

Promising results were also shown with the combination of group antenatal care (gANC: sessions for groups of women at similar stages of pregnancy) and individual check‐ups, with language support and integrated childbirth and parenting education offered to Somali women in Sweden [67]. In fact, despite the rate of high satisfaction (‘always happy with care’ rating) being similar between the intervention and control groups (OR 1.42, 95% CI 0.50–4.16), the former group showed better knowledge of danger signs (p 0.004) [67].

On the contrary, the Centering Pregnancy Model (CPM), which aimed to provide care and support during pregnancy and postpartum with bi‐weekly meetings, group discussions, educational components and risk assessments, was found to have no impact on the prenatal‐care knowledge, self‐esteem scores, or pregnancy‐related health behaviours measured with different scales among Hispanic women in the USA [53]. Similarly, the Asian Mother and Baby Campaign, undertaken with Asian women in the UK, showed no differences in the mean number of antenatal visits, admissions in pregnancy, gestational age at delivery, mode of delivery, in women who received visits and support from the link workers [59, 63]. A slight improvement in health service use in women with a good understanding of English was reported [63]. However, the unclear presentation of the aforementioned results might lower the quality of this evidence. No significant improvements in the women's satisfaction were recorded after the implementation of the community‐based bilingual doula (CBD) support in labour and postpartum among Somali, Arabic, Russian, Polish and Tigrinya women in Sweden (OR of ‘very happy with care’ 1.07, 95% CI 0.48–2.40) [66]. Similarly, no differences were noted in any of the maternal and neonatal outcomes [66].

### 3.3 Summary

**FIGURE 2:**
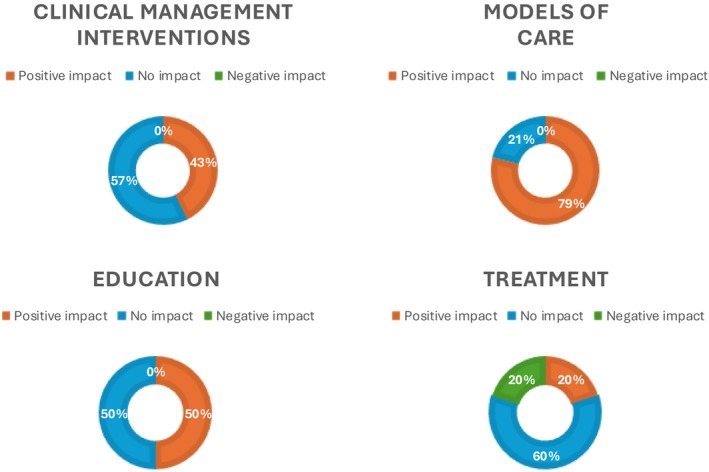
Impact of different interventions.

Overall, the majority of interventions have demonstrated a positive impact on improving maternal and perinatal care in these ethnic groups (21 out of 37 assessments, 56.7%) (Figure 2). Only one study demonstrated a negative impact of the intervention. The remaining studies showed no effect of the intervention on the outcomes of the study.

**FIGURE 3:**
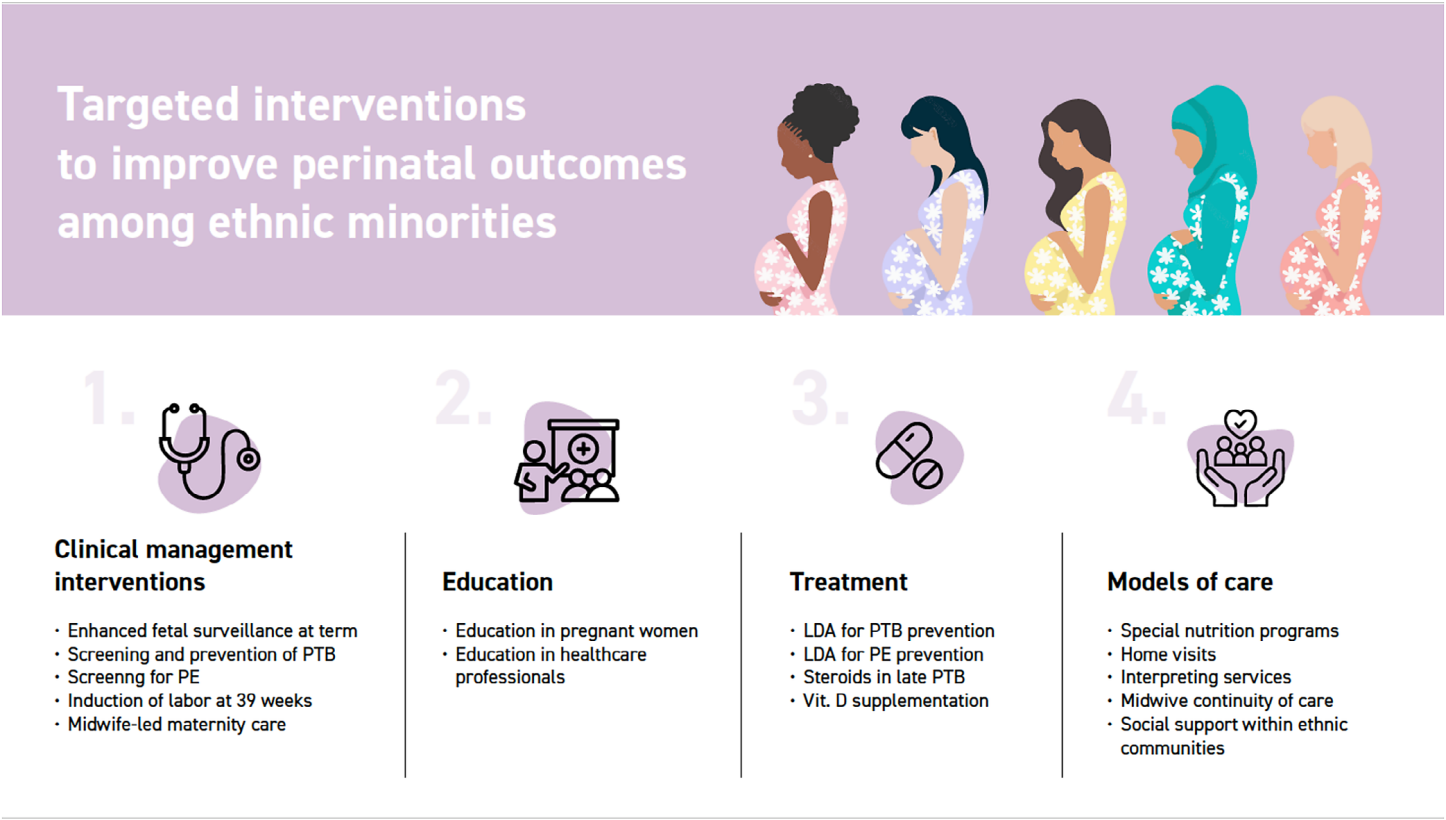
Infographic of interventions.

## 4 Discussion

### 4.1 Summary of Key Findings

The systematic review identified a range of interventions aimed at improving pregnancy outcomes among ethnic minority pregnant women, including changes in clinical practice, educational programmes, specific treatments and social support initiatives. However, a quantitative synthesis of results was not feasible due to heterogeneity in interventions and outcome measures. To analyse the interventions studied, we categorised them into four broad groups. First, clinical management interventions such as foetal surveillance and induction of labour showed mixed results. Whilst some studies reported significant reductions in the rates of stillbirth and neonatal deaths [32], others found no significant improvements in perinatal outcomes [37, 54]. Second, educational interventions and programmes targeting healthcare providers and patients [30, 31, 59] demonstrated some positive outcomes. Third, specific treatments, such as LDA for PTB prevention [36] and Vitamin D supplementation [65], showed mixed results, with some studies reporting positive effects while others showed limited impact [36, 43]. Models of care implementation initiatives were associated with reduced rates of stillbirth and PTB among specific ethnic groups [44, 52, 55]. Among First Nations/Aboriginal communities in Australia, different social support initiatives for pregnant women showed promising results [40, 49, 50, 51, 56, 57, 58, 60, 61].

### 4.2 Interpretation of Findings

Our findings align with existing literature, which highlights the lack of existing targeted interventions to improve outcomes in women who belong to ethnic minorities, particularly with a large number of studies focusing on the disparities faced by the Aboriginal/Indigenous communities. Studies have consistently shown that these women, particularly Black and Asian women, face higher risks of adverse pregnancy outcomes such as preterm birth, low birthweight and neonatal mortality [68]. In fact, in an analysis of 1,155,981 women in England, the impact of significant socio‐economic and ethnic disparities in adverse pregnancy outcomes has been demonstrated [69], revealing that 23.6% of stillbirths, 18.5% of preterm births and 31.1% of FGR cases were attributed to socio‐economic inequality, with the highest risks in Black and South Asian women [69]. A recent systematic review evaluating health and social care interventions targeting disadvantaged populations in high‐income countries synthesised findings from 46 studies from Australia, Canada, Chile, Hong Kong, the UK and the USA [28]. The latter identified three main intervention types: midwifery models of care, interdisciplinary care and community‐centred services. These interventions positively impacted maternal, perinatal and infant outcomes, as well as care quality and access, with different degrees of significance [28]. Compared to this systematic review, which included a broader spectrum of disadvantaged populations of pregnant women, our focus is specifically on ethnic minority pregnant women.

The National Health Service Race and Health Observatory (NHS RHO) report focuses on mapping existing policy interventions aimed at addressing ethnic health inequalities in maternal and perinatal outcomes [70]. It primarily uses a scoping review methodology, whereas we have performed a standard systematic review of international studies [70]. The NHS RHO report highlights several interventions that show promise, such as midwifery‐led continuity of care and health advocacy programs, but notes limitations due to the predominance of single‐site observational studies and qualitative research, which often lack adjustment for confounders and detailed policy analysis [70]. The findings of our review build upon the NHS RHO findings by providing a more comprehensive analysis of specific interventions across multiple countries.

Studies have consistently highlighted that it is incorrect to group all ethnicities together, as some interventions may appear more beneficial for one ethnic group while not as effective for others [38, 44, 64, 70]. This was also evident in our review, where a particular intervention, for example, low‐dose aspirin, was found to significantly reduce the risk of pre‐eclampsia in non‐White pregnant women, but the stratification for ethnic group showed significance among the Asian women, but not in the Black or Mixed/Other ethnic minority groups [38].

### 4.3 Clinical and Research Implications

Several studies have examined the implementation of midwifery‐led care among ethnic minority women as an intervention. Despite the heterogeneity in study populations and objectives, investing in such interventions may be justified, as midwifery care is generally more accessible than consultant‐led care across various countries. Given the barriers to healthcare access faced by ethnic minorities, including socio‐economic and language‐related challenges, midwifery‐led models could offer significant benefits in improving maternal and neonatal outcomes. In addition, given the promising results that some models of care have demonstrated, it is reasonable to promote the implementation of these initiatives in a public service like the NHS, where the continuity of care led by midwives, group antenatal care and support offered to ethnic minorities with language difficulties in the form of transportation and interpreting facilities would face the challenge of cultural and language barriers that are frequently seen in the UK. Community midwives, for instance, could devote a body of staff to the development of these services.

Educational interventions for pregnant women are based on the premise that active engagement in pregnancy can reduce morbidity through closer surveillance and timely intervention. Additionally, targeted education for healthcare providers on the care of ethnic minorities has the potential to enhance service quality for these populations. While existing studies have not conclusively demonstrated these benefits, further research is needed to identify and refine educational programmes that can effectively improve outcomes in these groups. In fact, increasing the awareness of these groups of women towards some obstetric complications might reduce the missed diagnoses and interventions because of lack of hospital attendance.

Finally, the literature has widely explored the implementation of social initiatives to improve outcomes among ethnic minorities, particularly in specific groups such as Indigenous Australians. These interventions have shown promising results, suggesting that social support programmes may be among the most effective strategies for enhancing pregnancy care in ethnic minority populations. As such, larger prospective studies are needed to further evaluate their impact and scalability.

The challenges that most clinical settings would face in the process of implementing initiatives to improve outcomes in ethnic minorities include the sensitisation of all healthcare providers to the different needs and requirements that these populations have, and the large‐scale cost‐effectiveness, which represents a major limitation, especially in public settings.

### 4.4 Strengths, Limitations and Future Directions

The review included a diverse range of studies from different countries and healthcare settings, providing a comprehensive overview of interventions targeting ethnic minority pregnant women. The high heterogeneity among the included studies and the limited number of studies on similar interventions, outcomes and populations hindered the ability to perform a meta‐analysis, making it difficult to generalise the findings and highlight the need for further studies in this area. Moreover, most studies do not establish a baseline, making it challenging to determine whether any observed positive changes are attributable to the intervention or other contextual factors that could influence maternal health. Many studies had small sample sizes and lacked control groups, which may affect the robustness of the conclusions. The review relied on published studies, which may be subject to publication bias. Additionally, most studies describe the interventions and their outcomes, but do not elucidate the implementation pathway or the challenges encountered in the implementation, which is crucial for determining the success of an intervention. The limitation of this review is that it exclusively includes studies from high‐income countries, which may not fully represent the global context, particularly in low‐income countries where different challenges and healthcare dynamics may exist. Future reviews may wish to consider broadening the scope of this review or replicating it in other aspects of perinatal healthcare [65, 66].

## 5 Conclusions

In conclusion, our systematic review highlights the variability and context dependence of interventions aimed at improving pregnancy outcomes among ethnic minority pregnant women. While educational and social support programmes show promise, further large‐scale, high‐quality studies are needed to evaluate and optimise these interventions.

## Author Contributions

S.S. conceptualization, writing and editing; S.P. conceptualization, writing and editing; N.E. data curation, metodology; F.F. data curation; L.A.M. conceptualization, editing, validation, writing; P.D. editing, validation, writing; S.A.S. editing, validation, conceptualization, writing; J.A. editing, validation, writing; S.T. editing, validation, writing; A.K. conceptualization, editing, supervision, writing.

## Conflicts of Interest

The authors declare no conflicts of interest.

## Supporting information


**Appendix S1:** bjo70013‐sup‐0001‐AppendixS1.docx.

## Data Availability

The data that support the findings of this study are available from the corresponding author upon reasonable request.

## References

[1] “MBRRACE‐UK: Mothers and Babies: Reducing Risk Through Audits and Confidential Enquiries Across the UK | MBRRACE‐UK | NPEU,” accessed July 13, 2024, https://www.npeu.ox.ac.uk/mbrrace‐uk.

[2] “Perinatal Mortality Surveillance | MBRRACE‐UK | NPEU,” accessed July 13, 2024, https://www.npeu.ox.ac.uk/mbrrace‐uk/reports/perinatal‐mortality‐surveillance#perinatal‐mortality‐for‐births‐2013‐supplementary‐report.

[3] “FIVEXMORE,” accessed July 13, 2024, https://fivexmore.org.

[4] T. Awe , C. Abe , M. Peter , and R. Wheeler , The Black Maternity Experiences Survey: A Nationwide Study of Black Women's Experiences of Maternity Services in the United Kingdom (Five X More, 2022).

[5] “Birthrights. Systemic Racism, Not Broken Bodies,” An Inquiry Into Racial Injustice and Human Rights in UK Maternity Care: Executive Summary, Coventry: Birthrights, 2022.

[6] S. Gohir , Invisible. Maternity Experiences of Muslim Women From Racialised Minority Communities (Muslim Women's Network UK, 2022).

[7] G. Kayode , B. Thilaganathan , C. Burden , et al., “Disparities in Stillbirths in England: Analysis of A Population‐Based Study of 1.3 Million Births,” BJOG: An International Journal of Obstetrics and Gynaecology 132, no. 8 (2025): 1130–1138. 40376868 10.1111/1471-0528.18147 PMC12137752

[8] E. E. Petersen , N. L. Davis , D. Goodman , et al., “Racial/Ethnic Disparities in Pregnancy‐Related Deaths — United States, 2007–2016,” MMWR. Morbidity and Mortality Weekly Report 68, no. 35 (2019): 762–765. 31487273 10.15585/mmwr.mm6835a3 PMC6730892

[9] A. A. Adane , B. M. Farrant , R. Marriott , S. W. White , H. D. Bailey , and C. C. J. Shepherd , “Socioethnic Disparities in Severe Maternal Morbidity in Western Australia: A Statewide Retrospective Cohort Study,” BMJ Open 10, no. 11 (2020): e039260. 10.1136/bmjopen-2020-039260 PMC7643510 33148750

[10] C. Maxwell , M. Tunde‐Byass , and K. Wilson‐Mitchell , “Achieving Equity in Reproductive Care and Birth Outcomes for Black People in Canada,” Canadian Medical Association Journal 196, no. 10 (2024): E343–E345. 38499307 10.1503/cmaj.231105 PMC10948181

[11] Q. Miao , Y. Guo , E. Erwin , et al., “Racial Variations of Adverse Perinatal Outcomes: A Population‐Based Retrospective Cohort Study in Ontario, Canada,” PLoS One 17, no. 6 (2022): e0269158. 35772371 10.1371/journal.pone.0269158 PMC9246499

[12] C. Diguisto , M. Saucedo , A. Kallianidis , et al., “Maternal Mortality in Eight European Countries With Enhanced Surveillance Systems: Descriptive Population Based Study,” BMJ 16 (2022): e070621. 10.1136/bmj-2022-070621 PMC9667469 36384872

[13] C. Winsloe , J. Elhindi , M. C. Vieira , et al., “Differences in Factors Associated With Preterm and Term Stillbirth: A Secondary Cohort Analysis of the DESiGN Trial,” BJOG: An International Journal of Obstetrics and Gynaecology 132, no. 1 (2025): 89–98. 39291344 10.1111/1471-0528.17951 PMC11612614

[14] D. Geddes‐Barton , R. Goldacre , M. Knight , N. Vousden , and R. Ramakrishnan , “Ethnic Disparities in Severe Maternal Morbidity and the Contribution of Deprivation: A Population‐Based Causal Analysis,” BJOG: An International Journal of Obstetrics and Gynaecology (2025), Online ahead of print. 10.1111/1471-0528.18254 PMC12592783 40511482

[15] K. R. van Daalen , J. Kaiser , S. Kebede , et al., “Racial Discrimination and Adverse Pregnancy Outcomes: A Systematic Review and Meta‐Analysis,” BMJ Global Health 7, no. 8 (2022): e009227. 10.1136/bmjgh-2022-009227 PMC9344988 35918071

[16] L. Bridle , S. Bassett , and S. A. Silverio , “‘We Couldn't Talk to Her’: A Qualitative Exploration of the Experiences of UK Midwives When Navigating Women's Care Without Language,” International Journal of Human Rights in Healthcare 14, no. 4 (2021): 359–373.

[17] H. Rayment‐Jones , J. Harris , A. Harden , S. A. Silverio , C. F. Turienzo , and J. Sandall , “Project20: Interpreter Services for Pregnant Women With Social Risk Factors in England: What Works, for Whom, in What Circumstances, and How?,” International Journal for Equity in Health 20, no. 1 (2021): 233. 34689772 10.1186/s12939-021-01570-8 PMC8543874

[18] H. Lovell , S. A. Silverio , L. Story , E. Skelton , and J. Matthew , “Factors Which Influence Ethnic Minority Women's Participation in Maternity Research: A Systematic Review of Quantitative and Qualitative Studies,” PLoS One 18, no. 2 (2023): e0282088. 36827386 10.1371/journal.pone.0282088 PMC9956875

[19] S. A. Silverio , N. Varman , Z. Barry , et al., “Inside the ‘Imperfect Mosaic’: Minority Ethnic Women's Qualitative Experiences of Race and Ethnicity During Pregnancy, Childbirth, and Maternity Care in the United Kingdom,” BMC Public Health 23, no. 1 (2023): 2555. 38129856 10.1186/s12889-023-17505-7 PMC10734065

[20] M. Knight , K. Bunch , N. Vousden , et al., “A National Cohort Study and Confidential Enquiry to Investigate Ethnic Disparities in Maternal Mortality,” EClinicalMedicine 43 (2022): 101237. 34977514 10.1016/j.eclinm.2021.101237 PMC8683666

[21] S. A. Silverio , K. De Backer , T. Dasgupta , et al., “On Race and Ethnicity During a Global Pandemic: An ‘Imperfect Mosaic’ of Maternal and Child Health Services in Ethnically‐Diverse South London, United Kingdom,” EClinicalMedicine 48 (2022): 101433. 35783482 10.1016/j.eclinm.2022.101433 PMC9249549

[22] The Lancet Digital Health , “Pregnancy in a Pandemic: Inequalities in Maternal Health,” Lancet Digit Health 4, no. 2 (2022): e75. 35090676 10.1016/S2589-7500(22)00005-X PMC8789237

[23] B. Hussain , A. Latif , S. Timmons , K. Nkhoma , and L. B. Nellums , “Overcoming COVID‐19 Vaccine Hesitancy Among Ethnic Minorities: A Systematic Review of UK Studies,” Vaccine 40, no. 25 (2022): 3413–3432. 35534309 10.1016/j.vaccine.2022.04.030 PMC9046074

[24] L. A. Magee , E. Molteni , V. Bowyer , et al., “National Surveillance Data Analysis of COVID‐19 Vaccine Uptake in England by Women of Reproductive Age,” Nature Communications 14, no. 1 (2023): 956. 10.1038/s41467-023-36125-8 PMC9947170 36813760

[25] H. Blakeway , S. Prasad , E. Kalafat , et al., “COVID‐19 Vaccination During Pregnancy: Coverage and Safety,” American Journal of Obstetrics and Gynecology 226, no. 2 (2022): 236.e1–236.e14. 10.1016/j.ajog.2021.08.007 PMC8352848 34389291

[26] D. Devakumar , S. S. Bhopal , and G. Shannon , “COVID‐19: The Great Unequaliser,” Journal of the Royal Society of Medicine 113, no. 6 (2020): 234–235. 32521203 10.1177/0141076820925434 PMC7370648

[27] C. Fernandez Turienzo , M. Newburn , A. Agyepong , et al., “Addressing Inequities in Maternal Health Among Women Living in Communities of Social Disadvantage and Ethnic Diversity,” BMC Public Health 21, no. 1 (2021): 176. 33478445 10.1186/s12889-021-10182-4 PMC7817762

[28] Z. Khan , Z. Vowles , C. Fernandez Turienzo , et al., “Targeted Health and Social Care Interventions for Women and Infants Who Are Disproportionately Impacted by Health Inequalities in High‐Income Countries: A Systematic Review,” International Journal for Equity in Health 22, no. 1 (2023): 131. 37434187 10.1186/s12939-023-01948-w PMC10334506

[29] D. Moher , “Preferred Reporting Items for Systematic Reviews and Meta‐Analyses: The PRISMA Statement,” Annals of Internal Medicine 151, no. 4 (2009): 264. 19622511 10.7326/0003-4819-151-4-200908180-00135

[30] “Newcastle‐Ottawa Scale for Assessing the Quality of Nonrandomised Studies in Meta‐Analyses,” http://www.ohri.ca/programs/clinical_epidemiology/oxford.asp. 10.1007/s10654-010-9491-z 20652370

[31] B. W. Mol , S. Lai , A. Rahim , et al., “Checklist to Assess Trustworthiness in Randomised Controlled Trials (TRACT Checklist): Concept Proposal and Pilot,” Research Integrity and Peer Review 8, no. 1 (2023): 6. 37337220 10.1186/s41073-023-00130-8 PMC10280869

[32] M. L. Davies‐Tuck , M. A. Davey , R. L. Hodges , and E. M. Wallace , “Fetal Surveillance From 39 Weeks' Gestation to Reduce Stillbirth in South Asian‐Born Women,” American Journal of Obstetrics and Gynecology 229, no. 3 (2023): 286.e1–286.e9. 10.1016/j.ajog.2023.02.028 36907532

[33] P. Muller , A. M. Karia , K. Webster , et al., “Induction of Labour at 39 Weeks and Adverse Outcomes in Low‐Risk Pregnancies According to Ethnicity, Socioeconomic Deprivation, and Parity: A National Cohort Study in England,” PLoS Medicine 20, no. 7 (2023): e1004259. 37471395 10.1371/journal.pmed.1004259 PMC10358943

[34] T. Damsted Rasmussen , S. Fredsted Villadsen , A. V. Hansen , et al., “Effectiveness Evaluation of an Antenatal Care Intervention Addressing Disparities to Improve Perinatal Outcomes in Denmark: A Nationwide Register‐Based Analysis of a Cluster Randomised Controlled Trial (MAMAACT),” BJOG: An International Journal of Obstetrics and Gynaecology 130, no. 7 (2023): 759–769. 36655509 10.1111/1471-0528.17404

[35] T. D. Rasmussen , A. M. Nybo Andersen , C. T. Ekstrøm , S. S. Jervelund , and S. F. Villadsen , “Improving Health Literacy Responsiveness to Reduce Ethnic and Social Disparity in Stillbirth and Infant Health: A Cluster Randomized Controlled Effectiveness Trial of the MAMAACT Intervention,” International Journal of Nursing Studies 144 (2023): 104505. 37267853 10.1016/j.ijnurstu.2023.104505

[36] V. A. Kane , M. Andrikopoulou , C. Bertozzi‐Villa , J. Mims , K. Pinson , and C. Gyamfi‐Bannerman , “Low‐Dose Aspirin and Racial Disparities in Spontaneous Preterm Delivery in Low‐Risk Individuals,” AJOG Global Reports 3, no. 4 (2023): 100273. 38034022 10.1016/j.xagr.2023.100273 PMC10682009

[37] Y. E. Berman , J. P. Newnham , S. W. White , K. Brown , and D. A. Doherty , “The Western Australian Preterm Birth Prevention Initiative: A Whole of State Singleton Pregnancy Cohort Study Showing the Need to Embrace Alternative Models of Care for Aboriginal Women,” BMC Pregnancy and Childbirth 23, no. 1 (2023): 7. 36600220 10.1186/s12884-022-05222-9 PMC9811788

[38] B. Liu , U. Nadeem , A. Frick , M. Alakaloko , A. Bhide , and B. Thilaganathan , “Reducing Health Inequality in Black, Asian and Other Minority Ethnic Pregnant Women: Impact of First Trimester Combined Screening for Placental Dysfunction on Perinatal Mortality,” BJOG: An International Journal of Obstetrics and Gynaecology 129, no. 10 (2022): 1750–1756. 35104381 10.1111/1471-0528.17109 PMC9544950

[39] R. Hadebe , P. T. Seed , D. Essien , et al., “Can Birth Outcome Inequality Be Reduced Using Targeted Caseload Midwifery in a Deprived Diverse Inner City Population? A Retrospective Cohort Study, London, UK,” BMJ Open 11, no. 11 (2021): e049991. 10.1136/bmjopen-2021-049991 PMC8562498 34725078

[40] S. Kildea , Y. Gao , S. Hickey , et al., “Effect of a Birthing on Country Service Redesign on Maternal and Neonatal Health Outcomes for First Nations Australians: A Prospective, Non‐Randomised, Interventional Trial,” Lancet Global Health 9, no. 5 (2021): e651–e659. 33743199 10.1016/S2214-109X(21)00061-9

[41] M. Andrikopoulou , U. N. Emeruwa , E. Ludwig , E. Overton , and C. Gyamfi‐Bannerman , “Race and Neonatal Respiratory Morbidity in the Late Preterm Period,” American Journal of Obstetrics & Gynecology MFM 3, no. 5 (2021): 100408. 34058419 10.1016/j.ajogmf.2021.100408

[42] A. Akselsson , H. Lindgren , S. Georgsson , K. Pettersson , V. Skokic , and I. Rådestad , “Pregnancy Outcomes Among Women Born in Somalia and Sweden Giving Birth in the Stockholm Area – A Population‐Based Study,” Global Health Action 13, no. 1 (2020): 1794107. 32744184 10.1080/16549716.2020.1794107 PMC7480426

[43] M. C. Tolcher , H. Sangi‐Haghpeykar , H. Mendez‐Figueroa , and K. M. Aagaard , “Low‐Dose Aspirin for Preeclampsia Prevention: Efficacy by Ethnicity and Race,” American Journal of Obstetrics & Gynecology MFM 2, no. 4 (2020): 100184. 33345910 10.1016/j.ajogmf.2020.100184

[44] M. Angley , V. R. Thorsten , C. Drews‐Botsch , et al., “Association of Participation in a Supplemental Nutrition Program With Stillbirth by Race, Ethnicity, and Maternal Characteristics,” BMC Pregnancy and Childbirth 18, no. 1 (2018): 306. 30041624 10.1186/s12884-018-1920-0 PMC6056947

[45] C. S. Homer , N. Leap , N. Edwards , and J. Sandall , “Midwifery Continuity of Carer in an Area of High Socio‐Economic Disadvantage in London: A Retrospective Analysis of Albany Midwifery Practice Outcomes Using Routine Data (1997–2009),” Midwifery 48 (2017): 1–10. 28284877 10.1016/j.midw.2017.02.009

[46] P. Middleton , T. Bubner , K. Glover , et al., “‘Partnerships Are Crucial’: An Evaluation of the Aboriginal Family Birthing Program in South Australia,” Australian and New Zealand Journal of Public Health 41, no. 1 (2017): 21–26. 27868308 10.1111/1753-6405.12599

[47] C. Reeve , S. Banfield , A. Thomas , D. Reeve , and S. Davis , “Community Outreach Midwifery‐Led Model Improves Antenatal Access in a Disadvantaged Population,” Australian Journal of Rural Health 24, no. 3 (2016): 200–206. 26390849 10.1111/ajr.12249

[48] S. D. Tandon , F. Cluxton‐Keller , L. Colon , P. Vega , and A. Alonso , “Improved Adequacy of Prenatal Care and Healthcare Utilization Among Low‐Income Latinas Receiving Group Prenatal Care,” Journal of Women's Health 22, no. 12 (2013): 1056–1061. 10.1089/jwh.2013.4352 24117000

[49] S. Kildea , H. Stapleton , R. Murphy , N. B. Low , and K. Gibbons , “The Murri Clinic: A Comparative Retrospective Study of an Antenatal Clinic Developed for Aboriginal and Torres Strait Islander Women,” BMC Pregnancy and Childbirth 12, no. 1 (2012): 159. 23256901 10.1186/1471-2393-12-159 PMC3548737

[50] E. Murphy and E. Best , “The Aboriginal Maternal and Infant Health Service: A Decade of Achievement in the Health of Women and Babies in NSW,” New South Wales Public Health Bulletin 23, no. 4 (2012): 68. 22697102 10.1071/NB11051

[51] R. Wong , A. Herceg , C. Patterson , et al., “Positive Impact of a Long‐Running Urban Aboriginal Medical Service Midwifery Program,” Australian and New Zealand Journal of Obstetrics and Gynaecology 51, no. 6 (2011): 518–522. 21806587 10.1111/j.1479-828X.2011.01326.x

[52] I. Khanani , J. Elam , R. Hearn , C. Jones , and N. Maseru , “The Impact of Prenatal WIC Participation on Infant Mortality and Racial Disparities,” American Journal of Public Health 100, no. S1 (2010): S204–S209. 20147683 10.2105/AJPH.2009.168922 PMC2837444

[53] B. Robertson , D. M. Aycock , and L. A. Darnell , “Comparison of Centering Pregnancy to Traditional Care in Hispanic Mothers,” Maternal and Child Health Journal 13, no. 3 (2009): 407–414. 18465216 10.1007/s10995-008-0353-1

[54] F. Simonet , R. Wilkins , E. Labranche , et al., “Primary Birthing Attendants and Birth Outcomes in Remote Inuit Communities—A Natural ‘Experiment’ in Nunavik, Canada,” Journal of Epidemiology and Community Health (1978) 63, no. 7 (2009): 546–551. 10.1136/jech.2008.080598 PMC2956754 19286689

[55] N. Wells , T. Sbrocco , C. W. Hsiao , L. D. Hill , N. A. Vaughn , and B. Lockley , “The Impact of Nurse Case Management Home Visitation on Birth Outcomes in African‐American Women,” Journal of the National Medical Association 100, no. 5 (2008): 547–552. 18507207 10.1016/s0027-9684(15)31301-8 PMC3033408

[56] K. S. Panaretto , M. R. Mitchell , L. Anderson , et al., “Sustainable Antenatal Care Services in an Urban Indigenous Community: The Townsville Experience,” Medical Journal of Australia 187, no. 1 (2007): 18–22. 17605698 10.5694/j.1326-5377.2007.tb01109.x

[57] K. S. Panaretto , H. M. Lee , M. R. Mitchell , et al., “Impact of a Collaborative Shared Antenatal Care Program for Urban Indigenous Women: A Prospective Cohort Study,” Medical Journal of Australia 182, no. 10 (2005): 514–519. 15896179 10.5694/j.1326-5377.2005.tb00017.x

[58] E. Tursan d'Espaignet , M. Measey , M. Carnegie , and D. Mackerras , “Monitoring the ‘Strong Women, Strong Babies, Strong Culture Program’: The First Eight Years,” Journal of Paediatrics and Child Health 39, no. 9 (2003): 668–672. 14629497 10.1046/j.1440-1754.2003.00272.x

[59] L. V. Klerman , S. L. Ramey , R. L. Goldenberg , S. Marbury , J. Hou , and S. P. Cliver , “A Randomized Trial of Augmented Prenatal Care for Multiple‐Risk, Medicaid‐Eligible African American Women,” American Journal of Public Health 91, no. 1 (2001): 105–111. 11189800 10.2105/ajph.91.1.105 PMC1446489

[60] D. Mackerras , “Birthweight Changes in the Pilot Phase of the Strong Women Strong Babies Strong Culture Program in the Northern Territory,” Australian and New Zealand Journal of Public Health 25, no. 1 (2001): 34–40. 11297299 10.1111/j.1467-842x.2001.tb00547.x

[61] R. M. Smith , P. A. Smith , M. McKinnon , and M. Gracey , “Birthweights and Growth of Infants in Five Aboriginal Communities,” Australian and New Zealand Journal of Public Health 24, no. 2 (2000): 124–135. 10790931 10.1111/j.1467-842x.2000.tb00132.x

[62] L. Parsons and S. Day , “Improving Obstetric Outcomes in Ethnic Minorities: An Evaluation of Health Advocacy in Hackney,” Journal of Public Health Medicine 14, no. 2 (1992): 183–191. 1515202

[63] E. S. Mason , “The Asian Mother and Baby Campaign (The Leicestershire Experience),” Journal of the Royal Society of Health 110, no. 1 (1990): 1–4. 2107306 10.1177/146642409011000101

[64] G. McEnery and K. P. S. Rao , “The Effectiveness of Antenatal Education of Pakistani and Indian Women Living in This Country,” Child: Care, Health and Development 12, no. 6 (1986): 385–399. 3815751 10.1111/j.1365-2214.1986.tb00516.x

[65] J. D. Maxwell , L. Ang , O. G. Brooke , and I. R. F. Brown , “Vitamin D Supplements Enhance Weight Gain and Nutritional Status in Pregnant Asians,” BJOG: An International Journal of Obstetrics and Gynaecology 88, no. 10 (1981): 987–991. 10.1111/j.1471-0528.1981.tb01686.x 6793058

[66] E. Schytt , A. Wahlberg , A. Eltayb , N. Tsekhmestruk , R. Small , and H. Lindgren , “Community‐Based Bilingual Doula Support During Labour and Birth to Improve Migrant Women's Intrapartum Care Experiences and Emotional Well‐Being–Findings From a Randomised Controlled Trial in Stockholm, Sweden [NCT03461640],” PLoS One 17, no. 11 (2022): e0277533. 36399476 10.1371/journal.pone.0277533 PMC9674173

[67] M. Ahrne , U. Byrskog , B. Essén , E. Andersson , R. Small , and E. Schytt , “Group Antenatal Care Compared With Standard Antenatal Care for Somali‐Swedish Women: A Historically Controlled Evaluation of the Hooyo Project,” BMJ Open 13, no. 1 (2023): e066000. 10.1136/bmjopen-2022-066000 PMC9884917 36697050

[68] J. Sheikh , J. Allotey , T. Kew , et al., “Effects of Race and Ethnicity on Perinatal Outcomes in High‐Income and Upper‐Middle‐Income Countries: An Individual Participant Data Meta‐Analysis of 2 198 655 Pregnancies,” Lancet 400, no. 10368 (2022): 2049–2062. 36502843 10.1016/S0140-6736(22)01191-6

[69] J. Jardine , K. Walker , I. Gurol‐Urganci , et al., “Adverse Pregnancy Outcomes Attributable to Socioeconomic and Ethnic Inequalities in England: A National Cohort Study,” Lancet 398, no. 10314 (2021): 1905–1912. 34735797 10.1016/S0140-6736(21)01595-6

[70] NHS ‐ Race and Health Observatory , “Mapping Existing Policy Interventions to Tackle Ethnic Health Inequalities in Maternal and Neonatal Health in England: A Systematic Scoping Review With Stakeholder Engagement,” 2022, https://www.nhsrho.org/wp‐content/uploads/2022/12/RHO‐Mapping‐existing‐policy‐interventions_December‐2022.pdf.

