# Supplementary material for: Interventions to Address Disparities in Perinatal Outcomes by Ethnicity: A Systematic Review
Source: BJOG. 2025 Sep 23;132(13):2009–23. doi: 10.1111/1471-0528.70013 (PMC12592784; doi:10.1111/1471-0528.70013)
Supplement: Supplementary file 1 — Appendix S1: bjo70013‐sup‐0001‐AppendixS1.docx. [file BJO-132-2009-s001.docx]

**Supplementary material**

**Appendix S1. Search Strategies**

**Databases:** Embase®, MEDLINE®

(search strategy run and results downloaded on 2 February 2024)

| **Set#** | **Searched for** | **Results** |
| --- | --- | --- |
| S1 | MESH.EXACT("Stillbirth") OR MESH.EXACT("Fetal Death") OR MESH.EXACT("Perinatal Death") OR MESH.EXACT("Abortion, Habitual") OR MESH.EXACT("Abortion, Spontaneous") OR MESH.EXACT("Abortion, Missed") OR MESH.EXACT("Perinatal Mortality") OR MESH.EXACT("Fetal Mortality") | 61778* |
| S2 | EMB.EXACT("stillbirth") OR EMB.EXACT("fetus death") OR EMB.EXACT("perinatal death") OR EMB.EXACT("missed abortion") OR EMB.EXACT("recurrent abortion") OR EMB.EXACT("spontaneous abortion") OR EMB.EXACT("fetus wastage") OR EMB.EXACT("perinatal mortality") OR EMB.EXACT("fetus mortality") | 139685* |
| S3 | ti,ab,if(stillbirth or stillbirths or stillborn or stillborns or "still birth" or "still births" or "still born" or "still borns" or miscarriage* or IUFD) or ti,ab,if(pregnan* near/5 (loss or losses)) | 127639* |
| S4 | ti,ab,if((fetal or foetal or fetus or foetus or perinatal or "peri natal" or antenatal or "ante natal" or prenatal or "pre natal" or peripartum or "peri partum" or antepartum or "ante partum" or prepartum or "pre partum" or intrauterine or "intra uterine") near/5 (death or deaths or loss or losses or demise or mortalit*)) | 161754* |
| S5 | ti,ab,if((spontaneous or habitual or repeated or recurrent or missed) near/5 (aborted or abortion or abortions)) | 77671* |
| S6 | MESH.EXACT("Ethnicity") OR MESH.EXACT("Ethnic and Racial Minorities") OR MESH.EXACT("Racial Groups") OR MESH.EXACT("Race Factors") OR QU(eh) OR MESH.EXACT("Ethnology") | 249014* |
| S7 | EMB.EXACT("ethnicity") OR EMB.EXACT("ethnic background") OR EMB.EXACT("ethnic or racial aspects") OR EMB.EXACT("ethnic difference") OR EMB.EXACT("ethnic group") OR EMB.EXACT("ethnic identity") OR EMB.EXACT("racial background") OR EMB.EXACT("racial identity") OR EMB.EXACT("race difference") OR EMB.EXACT("race") OR EMB.EXACT("multiracial person") OR EMB.EXACT("ethnology") OR EMB.EXACT("ethnography") OR EMB.EXACT("ethnographic research") | 450234* |
| S8 | ti,ab,if(ethnic or ethnical or ethnicit* or racial or race or races or multiethnic* or multiracial or ethnolog* or ethnograph*) | 926326* |
| S9 | (s1 or s2 or s3 or s4 or s5) and (s6 or s7 or s8) | 10054* |
| S10 | rtype.exact("Clinical Trial, Phase III" OR "Randomized Controlled Trial" OR "Controlled Clinical Trial" OR "Clinical Trial, Phase IV" OR "Clinical Trial, Phase I" OR "Meta-Analysis" OR "Clinical Trial" OR "Clinical Trial, Phase II" OR "Pragmatic Clinical Trial" OR "Equivalence Trial" OR "Adaptive Clinical Trial" OR "Multicenter Study" OR "Systematic Review") | 1525702* |
| S11 | MESH.EXACT.EXPLODE("Clinical Trials as Topic") OR MESH.EXACT.EXPLODE("Meta-Analysis as Topic") OR MESH.EXACT("Random Allocation") OR MESH.EXACT("Single-Blind Method") OR MESH.EXACT("Cross-Over Studies") OR MESH.EXACT("Double-Blind Method") OR MESH.EXACT("Placebos") OR MESH.EXACT("Multicenter Studies as Topic") OR MESH.EXACT("Systematic Reviews as Topic") OR MESH.EXACT("Review Literature as Topic") | 741917* |
| S12 | EMB.EXACT("phase 1 clinical trial") OR EMB.EXACT("phase 3 clinical trial") OR EMB.EXACT.EXPLODE("randomized controlled trial") OR EMB.EXACT("phase 2 clinical trial") OR EMB.EXACT("phase 4 clinical trial") OR EMB.EXACT("controlled clinical trial") OR EMB.EXACT.EXPLODE("clinical trial") OR EMB.EXACT("triple blind procedure") OR EMB.EXACT("double blind procedure") OR EMB.EXACT("crossover procedure") OR EMB.EXACT("single blind procedure") OR EMB.EXACT.EXPLODE("randomization") OR EMB.EXACT("placebo") OR EMB.EXACT("multicenter study") OR EMB.EXACT.EXPLODE("meta analysis") OR EMB.EXACT("systematic review") OR EMB.EXACT.EXPLODE("clinical trial (topic)") OR EMB.EXACT("meta analysis (topic)") OR EMB.EXACT("systematic review (topic)") OR QU(ct) | 3217050* |
| S13 | ti,ab,if("meta analy[*3]" or metaanaly[*3] or "systematic review[*1]" or "systematic overview[*1]" or "integrative review[*1]" or "integrative research review[*1]" or "rapid review[*1]" or "umbrella review[*1]" or "scoping review[*1]" or "realist review[*1]" or trial[*1] or ((singl[*4] or doubl[*4] or trebl[*4] or tripl[*4]) near/5 (blind[*4] or mask[*4])) or rct[*1] or randomized or randomised or randomization or randomisation or randomly or crossover or "cross over" or placebo[*1] or (random near/3 allocat[*4])) | 6510549* |
| S14 | pub.exact("Cochrane Database of Systematic Reviews" OR "Cochrane database of systematic reviews (Online)" OR "The Cochrane database of systematic reviews") | 41401* |
| S15 | MESH.EXACT("Cohort Studies") OR MESH.EXACT.EXPLODE("Longitudinal Studies") OR MESH.EXACT("Follow-Up Studies") OR MESH.EXACT("Retrospective Studies") OR MESH.EXACT("Case-Control Studies") OR MESH.EXACT("Cross-Sectional Studies") OR MESH.EXACT("Prospective Studies") OR MESH.EXACT("Controlled Before-After Studies") OR MESH.EXACT("Historically Controlled Study") OR MESH.EXACT("Interrupted Time Series Analysis") OR MESH.EXACT("Epidemiologic Studies") OR MESH.EXACT("Pilot Projects") OR MESH.EXACT("Feasibility Studies") OR MESH.EXACT("Observational Studies as Topic") OR MESH.EXACT("Clinical Studies as Topic") | 3382755* |
| S16 | rtype.exact("Observational Study" or "Comparative Study" or "Clinical Study") | 2059028* |
| S17 | EMB.EXACT.EXPLODE("longitudinal study") OR EMB.EXACT.EXPLODE("case control study") OR EMB.EXACT("prospective study") OR EMB.EXACT("major clinical study") OR EMB.EXACT("retrospective study") OR EMB.EXACT("clinical study") OR EMB.EXACT("cross-sectional study") OR EMB.EXACT("cohort analysis") OR EMB.EXACT("observational study") OR EMB.EXACT("community trial") OR EMB.EXACT("open study") OR EMB.EXACT("family study") OR EMB.EXACT("pilot study") OR EMB.EXACT("comparative study") OR EMB.EXACT("controlled study") OR EMB.EXACT("follow up") OR EMB.EXACT("evaluation and follow up") | 15732912* |
| S18 | ti,ab,if(prospective[*2] or retrospective[*2] or "follow up" or followup or longitudinal[*2] or "case control[*4]" or "cross sectional[*2]" or observation[*2] or "population based" or "population study" or "population studies" or (case[*1] near/5 series) or "consecutive cases") or ti,ab((cohort or epidemiologic[*4]) near/5 (study or studies or analy[*3])) or ti(cohort) | 12380165* |
| S19 | (s1 or s2 or s3 or s4 or s5) and (s6 or s7 or s8) and (s10 or s11 or s12 or s13 or s14 or s15 or s16 or s17 or s18) | 7053* |
| **S20** | **(s1 or s2 or s3 or s4 or s5) and (s6 or s7 or s8) and (s10 or s11 or s12 or s13 or s14 or s15 or s16 or s17 or s18) and pd(2014-2024)** | **2985°** |
| **S21** | **((s1 or s2 or s3 or s4 or s5) and (s6 or s7 or s8) and (s10 or s11 or s12 or s13 or s14 or s15 or s16 or s17 or s18)) not pd(2014-2024)** | **2254°** |

* Duplicates are removed from the search, but included in the result count.

° Duplicates are removed from the search and from the result count.

**Databases:** Cochrane Library®

(search strategy run and results downloaded on 2 February 2024)

| **ID** | **Search** | **Hits** |
| --- | --- | --- |
| #1 | MeSH descriptor: [Stillbirth] this term only | 233 |
| #2 | MeSH descriptor: [Fetal Death] this term only | 294 |
| #3 | MeSH descriptor: [Perinatal Death] this term only | 159 |
| #4 | MeSH descriptor: [Abortion, Habitual] this term only | 321 |
| #5 | MeSH descriptor: [Abortion, Spontaneous] this term only | 802 |
| #6 | MeSH descriptor: [Abortion, Missed] this term only | 93 |
| #7 | MeSH descriptor: [Perinatal Mortality] this term only | 148 |
| #8 | MeSH descriptor: [Fetal Mortality] this term only | 4 |
| #9 | (stillbirth or stillbirths or stillborn or stillborns or "still birth" or "still births" or "still born" or "still borns" or miscarriage* or IUFD):ti,ab,kw | 4327 |
| #10 | (pregnan* near/5 (loss or losses)):ti,ab,kw | 1398 |
| #11 | ((fetal or foetal or fetus or foetus or perinatal or "peri natal" or antenatal or "ante natal" or prenatal or "pre natal" or peripartum or "peri partum" or antepartum or "ante partum" or prepartum or "pre partum" or intrauterine or "intra uterine") near/5 (death or deaths or loss or losses or demise or mortalit*)):ti,ab,kw | 3520 |
| #12 | ((spontaneous or habitual or repeated or recurrent or missed) near/5 (aborted or abortion or abortions)):ti,ab,kw | 3754 |
| #13 | #1 or #2 or #3 or #4 or #5 or #6 or #7 or #8 or #9 or #10 or #11 or #12 | 9633 |
| #14 | MeSH descriptor: [Ethnicity] this term only | 1386 |
| #15 | MeSH descriptor: [Ethnic and Racial Minorities] this term only | 26 |
| #16 | MeSH descriptor: [Racial Groups] this term only | 677 |
| #17 | MeSH descriptor: [Race Factors] this term only | 45 |
| #18 | MeSH descriptor: [] explode all trees and with qualifier(s): [ethnology - EH] | 5427 |
| #19 | MeSH descriptor: [Ethnology] this term only | 9 |
| #20 | (ethnic or ethnical or ethnicit* or racial or race or races or multiethnic* or multiracial or ethnolog* or ethnograph*):ti,ab,kw | 27915 |
| #21 | #14 or #15 or #16 or #17 or #18 or #19 or #20 | 27915 |
| **#22** | **#13 and #21** | **244** |

- The Cochrane Database of Systematic Reviews (CDSR, Cochrane Reviews) (12 references)
- The Cochrane Central Register of Controlled Trials (CENTRAL, Trials) (232 references)

**Table S1. Excluded studies**

| Authors | Year | Title | Reason for exclusion |
| --- | --- | --- | --- |
| Ekezie et al. | 2024 | Perinatal health outcomes of women from Gypsy, Roma and Traveller communities: A systematic review | No outcome of interest |
| Keenan-Devlin et al. | 2023 | Inflammatory markers in serum and placenta in a randomized controlled trial of group prenatal care | Outcomes not addressed by ethnic group |
| Moore et al. | 2023 | The Association of Race and Ethnicity with Adverse Outcomes in Women Treated for Mild Chronic Hypertension during Pregnancy | Abstract only |
| Khan et al. | 2023 | Targeted health and social care interventions for women and infants who are disproportionately impacted by health inequalities in high-income countries: a systematic review | Review |
| Nguemeni et al. | 2023 | Is racial segregation of hospital services associated with successful vaginal birth after cesarean? | Only abstract |
| Dickson et al. | 2023 | Socio-economic disadvantage and quality Antenatal Care (ANC) in Sierra Leone: Evidence from Demographic and Health Survey | No population of interest |
| Dube et al. | 2023 | Effect of an Australian community-based caseload midwifery group practice service on maternal and neonatal outcomes for women from a refugee background | No population of interest |
| Goodwin et al. | 2022 | Addressing social inequity through improving relational care: A social-ecological model based on the experiences of migrant women and midwives in South Wales | No intervention |
| Arechvo et al. | 2022 | Maternal Race and Stillbirth: Cohort Study and Systematic Review with Meta-Analysis | Review |
| Petersen et al. | 2022 | Knowledge About How to Manage Warning Signs of Pregnancy Complications Among Immigrants and Their Descendants Compared to Women of Danish Origin | No intervention |
| Stacey et al. | 2021 | An exploration of migrant women’s perceptions of public health messages to reduce stillbirth in the UK: a qualitative study | No intervention |
| Green et al. | 2021 | The predicted clinical workload associated with early post-term surveillance and inductions of labour in south Asian women in a non-tertiary hospital setting | No intervention |
| Doshi et al. | 2021 | 1153 Influence of race on adverse perinatal outcomes in women undergoing TOLAC | Only abstract |
| Harrison et al. | 2021 | Audio-visual patient leaflets: Making information accessible to women with limited English | Only abstract |
| Doshi et al. | 2021 | Influence of race on adverse perinatal outcomes in women undergoing TOLAC | Only abstract |
| Rayment-Jones et al. | 2021 | Project20: interpreter services for pregnant women with social risk factors in England: what works, for whom, in what circumstances, and how? | No ethnic minority |
| Baldewsingh et al. | 2020 | Adequate antenatal care and ethnicity affect preterm birth in pregnant women living in the tropical rainforest of Suriname | No intervention |
| Turienzo et al. | 2020 | Midwifery continuity of care versus standard maternity care for women at increased risk of preterm birth: A hybrid implementation–effectiveness, randomised controlled pilot trial in the UK | No population of interest |
| Stone et al. | 2020 | Evaluation of pilot guidelines for earlier term pregnancy monitoring of women of south asian ethnicity | Only abstract |
| Ramachandran et al. | 2020 | Risk of late stillbirth for women of South Asian ethnicity: Is induction of labour at 40 weeks the way forward? | Only abstract |
| Johnsen et al. | 2020 | Addressing ethnic disparity in antenatal care: A qualitative evaluation of midwives' experiences with the MAMAACT intervention | No outcome of interest |
| Holliman et al. | 2020 | Implementation of hemorrhage guidelines reveals racial disparities in transfusion requirements | Only abstract |
| Filby et al. | 2020 | A service evaluation of a specialist migrant maternity service from the user’s perspective | No outcome of interest |
| Hirshberg et al. | 2019 | Text message remote monitoring reduced racial disparities in postpartum blood pressure ascertainment | Unclear data |
| Kildea et al. | 2019 | Reducing preterm birth amongst Aboriginal and Torres Strait Islander babies: A prospective cohort study, Brisbane, Australia | Same cohort already included |
| Sina et al. | 2018 | Development of an integrated, district-wide approach to pre-pregnancy management for women with pre-existing diabetes in a multi-ethnic population | Outcomes not addressed by ethnic group |
| Larkin et al. | 2018 | Effect of Population-Specific Birthweight Curves on Disparities in Perinatal Mortality in Small-for-Gestational Age Pregnancies | No intervention |
| Coté-Arsenault et al. | 2019 | African American and Latino bereaved parent health outcomes after receiving perinatal palliative care: A comparative mixed methods case study | No intervention |
| Anjanappa et al. | 2017 | Quality improvement programme to reduce antepartum stillbirth | Only abstract |
| Boone et al. | 2016 | Effects of community health interventions on under-5 mortality in rural Guinea-Bissau (EPICS): a cluster-randomised controlled trial | No ethnic minority |
| Poston et al. | 2015 | Effect of a behavioural intervention in obese pregnant women (the UPBEAT study):a multicentre, randomised controlled trial | Outcomes not addressed by ethnic group |
| NCT01868230 | 2013 | Lifestyle Intervention in Overweight and Obese Pregnant Hispanic Women | Study protocol |
| Weissgerber et al. | 2013 | Haptoglobin Phenotype, Preeclampsia Risk and the Efficacy of Vitamin C and E Supplementation to Prevent Preeclampsia in a Racially Diverse Population | No outcome of interest |
| Berggren et al. | 2012 | Perinatal outcomes in Hispanic and non-Hispanic white women with mild gestational diabetes | No intervention |
| Zhou et al. | 2012 | Effectiveness of an intervention on uptake of maternal care in four counties in Ningxia, China | No ethnic minority |
| Schlenker et al. | 2012 | The effect of prenatal support on birth outcomes in an urban midwestern county | Outcomes not addressed by ethnic group |
| Berggren et al. | 2012 | The NICHD-MFMU GDM study: Differences in perinatal outcomes between Hispanic women and Non-Hispanic White women with and without gestational diabetes | Only abstract |
| O’Shaughnessy et al. | 2012 | Sweet Mother: evaluation of a pilot mental health service for asylum-seeking mothers and babies | No cohort of interest |
| Barnes et al. | 2011 | Providing the family-nurse partnership programme through interpreters in England | Outcomes not addressed by ethnic group |
| McAree et al. | 2010 | Perceptions of group practice midwifery from women living in an ethnically diverse setting | Case series (<10); article not retrieved |
| Dhanaliwala et al. | 2009 | A comparative study of antenatal care received by women of ethnic minorities and White/British women in Bolton, UK | Only abstract |
| Tucker et al. | 2009 | The unbooked mother: A case-control study of maternal and fetal outcomes in a North London hospital | No ethnic minority |
| Muglu et al. | 2009 | Obstetric performance of multi-ethnic teenage pregnant women in Manchester attending dedicated antenatal service in a tertiary hospital | Only abstract |
| Power et al. | 2008 | Evaluation of the Southern Aboriginal Maternity Care Project | Case series |
| Healy et al. | 2006 | Early access to prenatal care: Implications for racial disparity in perinatal mortality | No intervention |
| Campbell et al. | 2004 | Maternity care with the Women’s Business Service at the Mildura Aboriginal Health Service | Outcomes not addressed by ethnic group |
| Jan et al. | 2004 | An holistic economic evaluation of an Aboriginal community-controlled midwifery programme in Western Sydney | No outcome of interest |
| Smith et al. | 2004 | Extending the role of the linkworker in weaning support. | No population of interest |
| Wiggins et al. | 2004 | The Social Support and Family Health Study: a randomised controlled trial and economic evaluation of two alternative forms of postnatal support for mothers living in disadvantaged inner-city areas | No ethnic minority |
| Modell et al. | 2000 | Informed choice in genetic screening for thalassaemia during pregnancy: audit from a national confidential inquiry | No outcome of interest |
| Fowler et al. | 1995 | Prenatal outreach: an approach to reduce infant mortality of African American infants | Only abstract |
| Goldenberg et al. | 1990 | The Alabama preterm birth prevention project | No ethnic minority |
| Modell et al. | 1984 | Effect of fetal diagnostic testing on birth-rate of thalassaemia major in Britain | No data about pregnancy outcome |

**Table S2. Quality assessment of included studies. NOS assessment for observational studies**.

| **Study** | **Selection** | **Comparability** | **Outcome** |
| --- | --- | --- | --- |
| Davies-Tuck et al 2023 | **** | ** | *** |
| Ahrne et al. 2023 | **** | ** | *** |
| Muller et al 2023 | *** | ** | *** |
| Rasmussen et al 2023 | **** | ** | ** |
| Rasmussen et al 2023 | **** | ** | ** |
| Berman et al 2023 | **** | ** | *** |
| Liu et al 2022 | **** | ** | *** |
| Hadebe et al 2021 | *** | ** | *** |
| Kildea et al 2021 | *** | ** | *** |
| Angley et al 2018 | **** | ** | ** |
| Homer et al 2017 | **** |  | *** |
| Middleton et al 2017 | **** | ** | ** |
| Reeve et al 2016 | **** | ** | *** |
| Tandon et al 2013 | **** | ** | ** |
| Wong et al 2011 | *** | * | ** |
| Kildea et al 2012 | *** | ** | *** |
| Murphy et al 2012 | *** | * | ** |
| Khanani et al 2010 | **** | * | *** |
| Robertson et al 2009 | **** | ** | *** |
| Simonet et al 2009 | **** | * | *** |
| Wells et al 2008 | *** | ** | *** |
| Panaretto et al 2007 | *** | * | *** |
| Panaretto et al 2005 | *** | ** | *** |
| D’Espaignet et al 2003 | ** | * | * |
| Mackerras et al 2001 | ** | * | * |
| Smith et al 2000 | ** | * | * |
| Parsons et al 1992 | **** | * | *** |
| Mason et al 1990 | *** | * | *** |
| McEnery et al 1986 | *** | ** | *** |

**Table S3. Quality assessment of included studies. TRACT screening for RCTs.**

| **Study** | **Governance** | **Author group** | **Plausibility of intervention usage** | **Timeframe** | **Drop-out rates** | **Baseline characteristics** | **Outcomes** |
| --- | --- | --- | --- | --- | --- | --- | --- |
| Kane et al 2023 | No concern | No concern | No concern | No concern | No concern | No concern | No concern |
| Schytt et al 2022 | No concern | No concern | No concern | No concern | No concern | No concern | No concern |
| Andrikopoulou et al 2021 | No concern | No concern | No concern | No concern | No concern | No concern | No concern |
| Akselsson et al 2020 | No concern | No concern | No concern | No concern | No concern | No concern | No concern |
| Tolcher et al 2020 | No concern | No concern | No concern | No concern | No concern | No concern | No concern |
| Klerman et al 2001 | No concern | No concern | No concern | No concern | No concern | No concern | No concern |
| Maxwell et al 1981 | Some concern | No concern | Some concern | No concern | No concern | No concern | No concern |

**Table S4**: Interventions and primary and secondary outcomes in the included studies

| **Study** | **Intervention** | **Primary outcome** | **Secondary outcomes** | **Results** |
| --- | --- | --- | --- | --- |
| Davies-Tuck et al. ^28^ | Biweekly cardiotocography and measurement of amniotic fluid from 39 weeks | Stillbirth rate (antepartum or intrapartum death) at 37 and at 39 weeks of gestation | Neonatal death at <7 days, admission to special care nursery, admission to neonatal intensive care, Apgar score <7 at 5 minutes, infant birthweight (g), gestational age at birth, IOL, and mode of birth | 64% reduction in the rate of stillbirth (aOR 0.36; 95% CI 0.13-0.9, p 0.47)  Reduced early neonatal death (3.1/1000 vs 1.3/1000; P=0.03) |
| Ahrne et al.^63^ | Combination of group antenatal care (gANC: sessions for groups of women at similar stages of pregnancy) and individual  check-ups, with language support and integrated childbirth and parenting education | Women’s overall ranking of the care received; Edinburgh Postnatal Depression Scale (EPDS) in pregnancy and post-partum | Women’s rating of all components of care; knowledge about danger sings in pregnancy; number of visits, development of obstetric complications (gestational diabetes, ..); birth outcomes (IOL, mode of delivery, blood loss, oxytocin) neonatal outcomes (gestational age at birth, birthweight, small for gestational age, large for gestational age, Apgar <7 at 5 minutes, admission to intensive care | 79% of women in the cANC group were “always happy” when interviewed at the end of pregnancy, and 21% were “mostly happy”. Women in the standard care group, were 95% always or mostly happy, whereas 5% were “sometimes happy”; this difference in the rate of “always happy” was not statistically significant: OR 1.42, 95% CI 0.50-4.16. Knowledge of danger signs was better in the gANC group (58% vs 28% in case of vaginal bleeding, p 0.004). Labor and birth outcomes were similar between the groups. |
| Muller et al. ^29^ | IOL at 39+0-39+6 weeks vs expectant management in low-risk pregnancies | Composite outcome including stillbirth, neonatal death within 28 days of birth, or presence of the English composite neonatal adverse outcome indicator (the E-NAOI) for liveborn babies | - | IOL was associated with reduction in adverse outcomes, however, no statistical differences were observed in the ethnic minority groups |
| Rasmussen et al. ^30^ | MAMAACT intervention: training program for midwives including 6-h training session in intercultural communication and cultural competence, two follow-up dialogue meetings, and health education materials for pregnant women on warning signs of pregnancy complications in six languages | A composite perinatal mortality and morbidity outcome: stillbirths, neonatal deaths, Apgar score <7, umbilical arterial pH < 7.0, admissions to neonatal intensive care unit (NICU) >48 h, and NICU admissions for mechanical ventilation | Each complication included in the composite outcome | The intervention determined a significant reduction in the rate of arterial pH <7 (aOR 0.27, 95% CI 0.09– 0.81) and a significant increase in the rate of NICU admission (aOR 1.36, 95%CI 1.05–1.76). The composite outcome was higher in the intervention group and lower if excluding NICU admission (but no statistically significant). The intervention had no effect on stillbirths, neonatal mortality, and NICU admissions for mechanical ventilation |
| Rasmussen et al. ^31^ | MAMAACT intervention | Active engagement  with healthcare providers; Navigating the healthcare system; Certainty of complications management | - | No effect of intervention in the “Active management” domain score (adjusted mean difference −0.07, 95 % CI (−0.23-0.09) or in the “Navigating the healthcare system” domain score (adjusted mean difference −0.04, 95 % CI −0.20–0.12); intervention caused higher awareness of symptoms, however no statistical significance was noted: redness, swelling, and heat in one leg: aOR 1.13 (95 % CI  0.73–1.73) and vaginal bleeding: aOR 1.75 (95 % CI 0.91–3.34). |
| Kane et al. ^32^ | Original RCT: administration of LDA (60 mg) for the prevention of preeclampsia in  low-risk individuals (13 to 25 weeks  of gestation without chronic conditions, including hypertension) | Spontaneous preterm delivery after preterm labor or preterm premature rupture of membranes at <34 weeks of gestation | PTB at <37 weeks, overall PTB (medically indicated and spontaneous) at <34 and <37 weeks of gestation | With White race as a reference,  Black race was associated  with higher risk of spontaneous PTB <37 weeks (OR 1.75; 95%CI 1.08−2.81; p 0.02), overall  PTB <34 weeks (OR,  2.46; 95% CI, 1.07−5.64; p 0.03), and overall PTB at <37 weeks (OR, 1.62; 95% CI, 1.06−2.49; p 0.03) in  unadjusted analyses. After adjustment for treatment, no longer evidence of these differences was noted. |
| Berman et al. ^33^ | Western Australia (WA) Initiative for singletons: measurement of cervix length at all mid-pregnancy morphology scans, administration of vaginal progesterone and/or cervical cerclage for women with a shortened cervix, administration of vaginal progesterone in case of history of spontaneous PTB, no iatrogenic delivery before 38+ weeks without medical or obstetric indication, provision of smoking cessation counselling to women who smoke, and a PTB Prevention Clinic at the tertiary center for high risk cases | Preterm birth rate overall and by gestational age (20–31 weeks and 32–36 weeks) | Preterm birth rate in tertiary and non-tertiary care centers, stillbirth rate | No improvements in the preterm birth rate in Aboriginal women (PTB rate increased from 31.0% in 2013 to 35.3% in 2019 (p=0.393)). |
| Liu et al. ^34^ | Fetal Medicine Foundation (FMF) screening for preeclampsia: algorithm-based risk assessment tool combining maternal history, mean arterial pressure (MAP), pregnancy-  associated plasma protein A (PAPP-A) and uterine artery pulsatility index (UtA PI). Pregnancies at high-risk of placental dysfunction were defined by a ≥1:50 chance of pre-term pre-eclampsia and received prophylactic low dose aspirin (150 mg once daily) until 36 weeks and serial growth ultrasound scans at 28 and 36 weeks, and were offered induction of labor at 40 weeks | Stillbirth, neonatal death and perinatal death rates | - | In the non-White group perinatal death rate dropped from 7.95 to 3.22/1000 births (OR 0.403, 95% CI 0.206–0.789). After stratification, statistical significance was only reached in the Asian group (OR 0.324, 95% CI 0.121-0.865); no significant reduction was registered in the Black ethnic group (0.558, 95% CI 0.187-1.666) or in the Mixed/Other ethnic group (0.351, 95% CI 0.058-2.114) |
| Schytt et al.^62^ | Community-based bilingual doula (CBD) support in labor and post-partum | Women’s care experience measured by a single item question taken from  the MFMCQ (Migrant Friendly Maternity Care Questionnaire): ‘In general, were you  happy with the healthcare you received?’ with response alternatives dichotomized into ‘Yes, very happy with care’ and ‘Less than very happy’ (Quite happy; Not very happy; No, not happy at all); EPDS evaluation | Overall satisfaction with labor support; maternal outcomes (IOL, CS, operative delivery, perineal injury, blood loss, episiotomy, analgesia); infant outcomes (Apgar score <7 at 5 minutes, perinatal death, breastfeeding) | No differences were registered in the overall women’s satisfaction (OR with “very happy with care” 1.07, 95% CI 0.48-2.40) or in any of maternal and perinatal outcomes. |
| Hadebe et al. ^35^ | Targeted caseload midwifery | Mode of delivery | Stillbirths, neonatal death, neonatal admission, gestation of birth, birth weight, breast feeding at time of birth and skin to skin contact; maternal morbidity including pregnancy induced hypertension, pre-eclampsia, gestational diabetes mellitus | Rate of Caesarean section was significantly reduced in women allocated to caseload midwifery compared with those allocated to traditional care (27.8% vs 43.1%; RR: 0.68; p=0.04; 95% CI 0.47 to 0.99) |
| Kildea et al. ^36^ | Birthing in Our Community (BiOC) service: continuity of care services provided by a caseload midwife; investments in the First Nations workforce; cultural activities; transport services; coordinated care between primary and tertiary care | Antenatal care attendance, smoking after 20 weeks, preterm birth, breastfeeding, discharge from hospital. | First antenatal visit in the first trimester, spontaneous labor, analgesia, mode of delivery, post-partum hemorrhage, physiological management of third stage of labor, perineal trauma, exclusive breastfeeding, loww birthweight, stillbirth, Apgar score <7 at 5 minutes, neonatal admission, neonatal death (within 28 days) | The intervention significantly reduced the rate of PTB (OR 0.42, 95% CI 0.26-0.66; p <0.0001), low birthweight (OR 0.60, 95% CI 0.41-0.89, p 0.012), admission to neonatal nursery (OR 0.69, 95% CI 0.51-0.92, p 0.013) and increased the rate of first visit in the first trimester (OR 1.35, 95% CI 1.05-1.73) and the number of visits (OR 1.49, 95% CI 1.06-2.09, p 0.021). No significant effects on smoking (p 0.40), breastfeeding (p 0.097), Caesarean section rate (p 0.86), stillbirth (p 0.83) and neonatal death (p 0.91). |
| Andrikopoulou et al. ^37^ | Original RCT: betamethasone for singleton pregnant women at high risk for late preterm delivery (34-36+5) (Antenatal Late Preterm Steroids, ALPS) | Neonatal composite outcome of treatment in the first 72 hours (continuous positive airway pressure or high-flow nasal cannula  >2 hours, oxygen >4 hours, extracorporeal membrane oxygenation or  mechanical ventilation) or stillbirth or neonatal death before 72 hours | Severe respiratory morbidity, respiratory distress syndrome, transient tachypnea of the newborn, apnea, neonatal intensive care unit admission, bronchopulmonary dysplasia, and surfactant administration | Race was not predictive of the primary outcome (need for respiratory support within 72 hours after birth) in the placebo or study group (betamethasone in late preterm). |
| Akselsson et al. ^38^ | Mindfetalness: practiced daily for about 15 min when the fetus is awake, the woman needs to lie down on the left side and monitors strength,  character, and frequency of the fetal movements without counting each movement.  Midwives leaflets given to Somali-born  women written in Somali | Apgar score of 0-6 (stillbirth  counting as zero) at 5 minutes | Apgar score <4 at 5 min, admission to neonatal intensive care unit (NICU), small for gestational age, mode of delivery, labor from gestation 41 + 6, preterm delivery | No statistically significant differences were noted in Apgar score <7 at 5 min (0.6% versus 2.7%,  aRR 0.22, 95% CI 0.01–1.11) and babies in need of transfer to NICU (4.7% versus 7.5%, aRR 0.54, 95% CI 0.24–1.08) in the intervention group. The rate of preterm delivery was significantly reduced in the intervention group (0.6% versus 3.1%, aRR 0.15, 95% CI 0.01–0.75) |
| Tolcher et al. ^39^ | Low-Risk Aspirin trial: normotensive, nulliparous women were randomized to 60 mg aspirin daily or placebo (between 13 and 26 weeks).  High-Risk Aspirin trial: women with pregestational insulin-treated diabetes mellitus, chronic hypertension, multiple gestations, or a history of preeclampsia were randomized to 60 mg aspirin  daily or placebo (between 13 and 26 weeks). | Preeclampsia | Gestational age at delivery,  preterm delivery, placental abruption, small for gestational age, stillbirth, and neonatal death | In the low-risk trial, the risk of preeclampsia was not different among the Hispanic, non-  Hispanic black, or other ethnicity groups. The risk of stillbirth was significantly increased among non-Hispanic black women who received aspirin compared with  non-Hispanic black women who received placebo (P 0.048).  In the high-risk trial, there was no reduction in the rate of preeclampsia in any ethnic group |
| Angley et al. ^40^ | WIC: provides assistance in purchasing nutritious foods to pregnant and postpartum women and infants and children up to five years of age | Stillbirth rate | - | WIC participation was associated with lower rate of stillbirth among non-Hispanic Black women (aOR: 0.34; 95% CI 0.16, 0.72) but not among Hispanic women (aOR: 0.91; 95% CI 0.52, 1.52) |
| Homer et al. ^41^ | Albany Midwifery Practice: two midwives assigned to each woman: a primary midwife responsible for  providing and coordinating her individualised care and a second  midwife. These two midwives provided antenatal, intrapartum and  postnatal care up to 28 days following birth | Complications during pregnancy, labor and birth; breastfeeding and neonatal outcomes; Apgar scores; birth weight; admission to the neonatal unit; and perinatal mortality | - | Perinatal mortality rate in BAME was lower than previously observed (less than 2.0 per 1000 births vs 8.8-9.8 per 1,000 reported in other studies) |
| Middleton et al. ^42^ | Aboriginal Family Birthing Program (AFBP) : provides education and training for health workers in prenatal, birthing and post-natal care; created intercultural partnership for skill exchange | Preterm birth (<37 weeks), low birthweight (<2500 gr), small for gestational age (<10^th^ centile) | - | No significant different were observed in the rate of preterm birth (p 0.57), low birthweight (0.41) or small for gestational age (0.88). |
| Reeve et al. ^43^ | Implementation of a midwifery-led interdisciplinary model of antenatal outreach care | Low birthweight (<2500 gr), preterm birth (<37 weeks), mode of delivery, intrauterine death | Quality of care indicators (number of scans during pregnancy, alcohol intake, smoking habit, infection screening) | No significant differences were observed in any of the perinatal outcomes. The intervention group had a significantly higher number of scans in pregnancy, and higher rate of women presenting for the first visit in the first trimester |
| Tandon et al. ^44^ | CenteringPregnancy group prenatal care model: two-hour prenatal care sessions offered to pregnant women as an education tool | Perceptions of prenatal care (assessed with the Patient Participation and Satisfaction Questionnaire (PPSQ)) | Active participation in prenatal care, satisfaction with time spent talking to the education provider, ability to talk to the provider in the preferred language, acceptability of prenatal visit waiting time, expected prenatal care visit ratio (observed /expected visits in pregnancy) | The study showed significant difference in satisfaction (mean satisfaction score 84.3 vs. 64.9, p < .001), active participation in prenatal care (mean participation score 39.7 vs. 28.1, p < .001), satisfaction with time spent talking with the provider (98% vs. 19%, p < .001), ability to talk to their prenatal care provider in the preferred language (99% vs. 19%, p < .001), acceptability of prenatal care visit waiting time (99% vs. 6%, p < .001), expected prenatal care visit ratio (101.9 vs 83.1, p <0.001) |
| Kildea et al. ^45^ | Murri Antenatal Clinic: clinical input provided by a hospital-employed Indigenous midwife and a non-Indigenous obstetrician; additional support available from Indigenous liaison officers who welcomed women and their families and helped them to feel comfortable, and provided support for women transferred in from rural and remote areas | Number of antenatal visits, PTB (<37 weeks), spontaneous onset of labor, vaginal delivery, Apgar <7 at 5 minutes, analgesia, perineal trauma, low birthweight, post-partum hemorrhage, NICU admission, breastfeeding | - | In the intervention group they observed a higher number women having 2-4 (15.8% vs 11%) or 5-7 (23.6 vs 19.7%) visits in pregnancy; however less cases had >8 visits (28.1% vs 37.7%) (p 0.007) and lower rates of NICU admission (15.9% vs 21.8%, p 0.036) were observed. Higher rates of first degree perineal trauma (96% vs 83.2%, p <0.001) were registered in the intervention group; no differences in any other perinatal outcome (5 minutes Apgar, onset of labor, mode of birth, preterm birth). |
| Murphy et al. ^46^ | Aboriginal Maternal and Infant Health Service: continuity-of-care model, with midwives and Aboriginal Health Workers collaboration for a culturally sensitive model, women centered, based on primary health-care principles and provided in partnership with Aboriginal people | Perinatal mortality, first visit before 20 weeks, low birthweight, preterm birth, breastfeeding rates | - | Reduced perinatal mortality rate (from 20.4 to 14.4 per 1000 births; no statistically significant); higher rates of first visit before 20 weeks (OR 1.2, 95%CI 1.01-1.4, p 0.03); lower rates of preterm delivery (OR 0.5, 95%CI 0.4-0.8, p <0.001). Non statistically significant improvement in breastfeeding rates and lower rates of low birthweight babies. |
| Wong et al. ^47^ | Aboriginal Midwifery Access Program (AMAP): full antenatal care from the first visit in pregnancy, including home visits, assistance with appointments for antenatal investigations and specialist care, transport, birth support, postnatal follow-up (two midwifes and one Aboriginal access worker) | Rates of caesarean sections, preterm deliveries, low birthweight babies, smoking during pregnancy | - | Slightly lower rates of Caesarean section (20% vs 27.6%), preterm delivery (18.8% vs 21.6%), low birthweight babies (18.8% vs 21%) were observed in the intervention group. No statistical analysis was performed. |
| Khanani et al. ^48^ | The Special Supplemental Nutrition Program for Women, Infants, and Children (WIC): provides supplemental food, nutrition counseling, and health services referrals for low-income pregnant women, post- partum mothers, and infants | Infant mortality rate (number of deaths to infants younger than 1 year per 1000 live births for the population selected), neonatal mortality (number of deaths to live-born infants through 28 days of life per 1000 live births for the population selected), preterm birth <37 and <34 weeks | - | African American women enrolled in WIC prenatally were much less likely to experience an infant death (9.6% vs 21%, p<0.001)  African American women who participated in WIC prenatally had lower rates of preterm birth (13.7% vs 20%, p < .001), both <37 (p<0.001) and <34 weeks (p<0.001) |
| Robertson et al. ^49^ | Centering Pregnancy Model (CPM): providing care and support for pregnancy and post-partum with sessions about risk assessment, didactic components and group discussion (biweekly meetings) | Satisfaction with care delivery model, health behaviors, prenatal/postnatal care knowledge, self-esteem and depression, breastfeeding initiation and continuation, infant birth weight, gestational age at delivery, mode of delivery and infant length of stay | - | No significant differences in pregnancy related health behaviors, prenatal care knowledge or self-esteem scores: only postnatally women in the traditional group had significantly higher self-esteem scores than mothers in the CPM group |
| Simonet et al.^50^ | Midwife-led maternity care | Perinatal death | Stillbirth, neonatal death, post-neonatal death, preterm, small-for-gestational-age and low birthweight | No statistically significant differences in the rates of perinatal death and other outcomes |
| Wells et al. ^51^ | Black Babies SMILE: nurses provide home visits minimum once a month. Needs of the patients determine the frequency of home visits | Preterm delivery and low birthweight rates | - | Home visitations reduced the rate of preterm birth (aOR 0.31, 95% CI 0.11–0.88); no effects observed in the rate of low birthweight (aOR 0.37, 95% CI 0.12–1.10) |
| Panaretto et al. ^52^ | Mums and Babies program: continuity of care, daily maternal and child health clinics, integrated team approach, family orientation, screening services, transport services, intervention for risk factors, others. | Antenatal visits per pregnancy, preterm birth, low birthweight, perinatal death | - | Higher numbers of antenatal visits (p <0.001) and lower rates of perinatal deaths (p 0.014) and preterm delivery (p 0.055) were observed in the intervention group; no differences in the rate of low birthweight (p 0.289). |
| Panaretto et al. ^53^ | Mums and Babies program: continuity of care, daily maternal and child health clinics, integrated team approach, family orientation, screening services, transport services, intervention for risk factors, others. | Preterm birth, low birthweight, small for gestational age, birthweight, perinatal death | Gestational age at first visit, rates of pregnancies with inadequate care, late first visit. | Significantly lower rates of preterm births (8.7% vs 14.3%, p 0.002) were observed in the intervention group. No differences in the rates of low birthweight (0.067), perinatal death (0.864). |
| D’Espaignet et al. ^54^ | Strong Women, Strong Babies, Strong Culture Program (SWSBSC): senior Aboriginal women to offer support ro young Aboriginal women to prepare for pregnancy, support during pregnancy, promotion of adoption of safe practices | Rates of low birthweight babies | - | Reduction in the rate of low birthweight babies (p 0.014) after implementation in one of the two Aboriginal communities (10.9% vs 15.3%). |
| Klerman et al. ^55^ | Augmented prenatal care (in Mother and Family Specialty Center) : educational support (ideal weight goal, daily vitamin assumption,..), smoking cessation program, social support, prenatal care following American College of Obstetrics and Gynecology guidelines, highly expert nurses in prenatal care, 2-weekly appointments, transport services, written and video materials to take home | Number of visits in pregnancy, participation to prenatal classes, prenatal care overall rating;  Rates of low birthweight, preterm birth, growth restriction, low Apgar score, stay in neonatal intensive care unit, Caesarean deliveries; mean gestational age at delivery | - | Significantly higher rates of prenatal care positive rating (94% vs 80%, p 0.002), higher number of prenatal visits (13.7+3.8 vs 11.9+3.8, p 0.001), higher participation in prenatal courses (79% vs 17%, p <0.001).  No significant differences were noted in perinatal outcomes (all p> 0.05). |
| Mackerras et al. ^56^ | Strong Women, Strong Babies, Strong Culture Program (SWSBSC): senior Aboriginal women to offer support to young Aboriginal women to prepare for pregnancy, support during pregnancy, promotion of adoption of safe practices | Birthweight, rate of low birthweight | - | The rate of low birthweight babies decreased significantly among the pilot communities of the implementation (19.8% vs 11.3%, p 0.02) |
| Smith et al. ^57^ | Two interventions:  Implementation of Strong Women, Strong Babies, Strong Culture Program (SWSBSC) and nutritional support intervention for pregnant women and mothers of young children | Preterm delivery, low birthweight | - | Lower rates of preterm delivery in the implementation group (14% vs 21.1%); similar rates of low birthweight (16.3% vs 15.2%). No statistical analysis was performed |
| Parsons et al. ^58^ | Multi-Ethnic Women's Health Project (MEWHP): interpreting service and aim to influence hospital policy and practice | Gestational age at delivery, livebirths, non-attendance rate, antenatal length of stay, antenatal investigations (amniocentesis, scan, aplhafetoprotein test), birthweight, low birthweight, onset of labor, mode of delivery | - | Significantly lower rate of elective Caesarean sections (2.3% vs 5.9%), higher rates of spontaneous delivery (86.8% vs 74.8%), lower rates of instrumental deliveries (6.6% vs 9.4%) and overall Caesarean sections (8.5% vs 17.4%); no significant differences in other outcomes |
| Mason et al. ^59^ | Asian Mother and Baby Campaign: link workers allocated to the hospital and to the community as interpreters and facilitators, and also for education | Obstetric outcomes,  patients’ use and understanding of health care during and after pregnancy, infants’ health | - | No differences in the mean number of antenatal visits, admissions in pregnancy, gestational age at delivery, mode of delivery, epidural rate; the study group had fewer stillbirths and slightly higher birthweight.  Slight improvement in the use of health service in women with a good understanding of English |
| McEnery et al. ^60^ | Antenatal education: a course of 12 weekly lectures about fertility, pregnancy, childbirth and child- rearing by a health visitor, midwife or nutritionist | Rate of obstetric interventions, infant birthweight, low birthweight, Apgar score at 1 minute, preterm birth, breastfeeding, 1-year assessment of infants | - | Higher rates of Caesarian births, continuous breastfeeding in the intervention group;  slightly lower rates of reduced infant growth at 1 year of age, higher rates of vaccinations and vitamin administration to the infants; no differences in Apgar scores or mean birthweight. |
| Maxwell et al. ^61^ | Vitamin D supplementation (1000 IU/day) | Maternal weight gain, neonatal assessment, protein measurements | - | Significantly higher weight gain the third trimester (p<0.001); higher concentration of thyroid binding prealbumin and retinol binding protein levels; no differences in mean gestational age or birthweight |

aOR: adjusted odds ratio; E-NAOI: is a validated outcome based on the presence of any of 15 neonatal diagnoses or 7 procedures recorded in a baby’s admission record in hospital statistics; NICU: neonatal intensive care unit ; IOL: induction of labor

**Table S5: Impact of targeted interventions on perinatal outcomes, stratified by ethnic minority groups**

|  | Black | Asian | First Nations/ Aboriginal | Mixed | Other | Unspecified or all non-majority group ethnicities considered together |
| --- | --- | --- | --- | --- | --- | --- |
| MATERNAL |  |  |  |  |  |  |
| PTB | LDA (60 mg) eliminated the ethnicity-associated increase in PTB, in a secondary analysis of RCT of LDA vs. placebo in preeclampsia prevention (N=1050 women)  [Kane]  The Special Supplemental Nutrition Program reduced the rate of PTB compared to that observed in non-participants (13.7% vs 20%, p <0.001), both <37 (p<0.001) and <34 weeks (p<0.001)  (N=5731 women)  [Khanani]  Nurse home visitations reduced the rate of preterm birth (aOR 0.31, 95% CI 0.11–0.88).  (N=48 women)  [Wells]  Augmented prenatal care showed no reduction in the rate of preterm birth (p 0.22)  (N=318 women)  [Kelman] |  | Comprehensive screening and management to reduce PTB: no improvements were observed in the preterm birth rate (PTB rate increased from 31.0% in 2013 to 35.3% in 2019 (p=0.393)). (N=19406 women)  [Berman]  Care provision by Inuit midwives (vs. Western physicians) did not affect the rate of PTB (aOR 0.94, 95% CI 0.73-1.20)  (N=1529 women) [Simonet]  The Aboriginal Family Birthing Program (AFBP) did not improve the rate of PTB among Aboriginal women in Australia  (p 0.57)  (N=486)  [Middleton]  The implementation of a midwifery-lead interdisciplinary model of care among Indigenous women in Australia showed no improvements in the rate of preterm birth (p 0.448).  (N=121 women)  [Reeve]  Aboriginal Midwifery Access Program (AMAP) determined slightly lower rates of preterm delivery (18.8% vs 21.6%) in the intervention group, with no statistical analysis.  (N=130) [Wong]  Mums and Babies program determined significantly lower rates of preterm births (8.7% vs 14.3%, p 0.002) in the intervention group. (N=456)  [Panaretto 2005] Similar results were observed in the cohort reported in 2007 by the same authors (p 0.05)  (N=781) [Panaretto 2007]  The intervention (Birth in Our Community service) significantly reduced the rate of PTB (OR 0.42, 95% CI 0.26-0.66; p <0.0001)  (N=766 women)  [Kildea 2021]  The Murri clinic did not influence the rate of PTB (p 0.364)  (N=367 women)  [Kildea 2012]  The Aboriginal Maternal and Infant Health Service determined a reduction in the rate of PTB (OR 0.5, 95%CI 0.4-0.8, p <0.001)  (N= 689)  [Murphy]  The implementation of the SWSBSC and of a nutritional support program showed a reduction in the rate of preterm delivery (14% vs 21.1%; no stastistical analysis was perfoemd).  (N=43)  [Smith] |  | Mindfetalness practice (active role of women in monitoring quality of fetal movement) reduced PTB (aRR 0.15 [0.01-0.75) (N=169 Somali women)  [Askelsson] | Case-loading midwifery-led care (vs. usual care) did not reduce PTB at <37 weeks (RR 0.49, 95% CI 0.21 to 1.09; p 0.08) or PTB at <34 weeks (RR 0.24, 95% CI 0.05 to 1.12; p 0.07) in BAME groups (N=110 women)  [Hadebe]  Compared to white women, BAME women assigned to two-midwifery care experienced higher rates of preterm birth (6.2% vs 3.2%; p<0.001). (N=1620)  [Homer] |
| Pre-eclampsia | LDA (60mg) did not affect PE in high- or low-risk non-Hispanic in the secondary analysis of two RCTs (low and high risk women for PE) (N=42 low-risk women, N=136 high risk women)  [Tolcher] |  |  |  | LDA 60mg did not affect PE in high- or low-risk Hispanic women or women of other ethnicity in the secondary analysis of two RCTs (low and high risk women for PE) (N=24 low-risk women; N=22 high-risk women)  [Tolcher] |  |
| Caesarean birth | Augmented prenatal care showed no reduction in the rate of Casarean deliveries (p 0.24)  (N=318 women)  [Kelman] | Multi-Ethnic Women's Health Project, consisting of interpreting services and changes in hospital policies reduced the rate of Caesarean sections and instrumental deliveries (p<0.001), increased the rates of spontaneous delivery (p 0.02)  In Asian and Turkish women.  (N=1000)  [Parsons]  The Asian Mother and Baby Campaign consisting of linkworkers, interpreters and facilitators in the hospitals and community determined no differences in the gestational age at delivery or mode of delivery.  (N=213 women)  [Mason]  General reproductive education was associated with more Caesareans (25% vs 15%) (N=69 women)  [McEnery] | The implementation of a midwifery-lead interdisciplinary model of care among Indigenous women in Australia showed no improvements in the rate of Caesarean section (0.507).  (N=121 women)  [Reeve]  Aboriginal Midwifery Access Program (AMAP) determined slightly lower rates of Caesarean section (20% vs 27.6%) in the intervention group, with no statistical analysis.  (N=130) [Wong]  The intervention (Birth in Our Community service) did not reduce the Caesarean section rate (p 0.86).  (N= 766 women)  [Kildea 2021] |  | The group ANC in the Somali-born women in Sweden caused no differences in the rates od CS (p 0.06)  [Ahrne] | Case-loading midwifery-led care (vs. usual care) reduced Caesareans (RR 0.68, 95% CI 0.47-0.99; p 0.04), including any Caesarean section (N=110 women) [Hadebe]  Compared to white women, BAME women assigned to two-midwifery care experienced higher rates of Caesarean delivery (4.4% vs 2.9%) (N=1620)  [Homer] |
| Weight-gain |  | Vitamin D supplementation was associated with higher weight gain in the third trimester (p<0.001) and higher concentrations of thyroid binding prealbumin and retinol binding protein levels (p 0.01)  (N=59 women)  [Maxwell] |  |  |  |  |
| PERINATAL |  |  |  |  |  |  |
| Stillbirth | Assistance in purchasing nutritious food decreased the rate of stillbirth (aOR: 0.34; 95% CI 0.16-0.72)  [Angley] | Enhanced fetal surveillance (pre-post comparison) reduced stillbirth (aOR 0.36 [0.13-0.90] (N=8532 women)  [Davies-Tuck] | Care provision by Inuit midwives (vs. Western physicians) did not affect stillbirth (aOR 0.97, 95% CI 0.39-2.41) or perinatal death (aOR 1.29, 95% CI 0.63-2.64)  (N=1529 women) [Simonet]  The implementation of a midwifery-lead interdisciplinary model of care among Indigenous women in Australia showed no improvements in the rate of  intrauterine death (p 0.489)  (N=121 women)  Reeve  The intervention (Birth in Our Community service) did not reduce the rate of stillbirth (p 0.83).  (N= 766 women)  (Kildea 2021) |  | Assistance in purchasing nutritious food did not decrease the rate of stillbirth in Hispanic women (aOR: 0.91; 95% CI 0.52-1.52)  [Angley]  The group ANC in the Somali-born women in Sweden caused no differences in the rates of stillbirth (p 1.000)  [Ahrne] | Screening algorithm for PE (vs. clinical risk factor screening) reduced stillbirth in non-White minority groups (OR 0.40 [0.21-0.79]) (N=4349 women)  [Liu]  Non-significant reduction was observed in the rate of stillbirth with elective IOL at 39 weeks in ethnic minorities (N=47.352 women)  [Muller]  Midwife education had no significant effect on the rate of stillbirth or neonatal death (aOR 0.88, 95% CI 0.40-1.94 and aOR 0.54, 95% CI 0.14–2.15)(N=14.835 women)  [Rasmussen 1] |
| SGA infants | Nurse home visitations did not influence the rate of low birthweight (aOR 0.37, 95% CI 0.12–1.10)  (N=48 women)  [Wells]  Augmented prenatal care showed no reduction in the rate of low birthweight (p 0.60)  (N=318 women)  [Kelman] | Vitamin D supplementation was not associated with differences in the mean birth weight  (N=59 women)  [Maxwell] | Care provision by Inuit midwives (vs. Western physicians) did not affect the rate of SGA (aOR 1.48, 95% CI 0.82-2.68)  (N=1529 women) [Simonet]  The Aboriginal Family Birthing Program (AFBP) did not improve the rate of SGA (p 0.88) or low birthweight (p 0.41)  among Aboriginal women in Australia  (N=486 women)  [Middleton]  The implementation of a midwifery-lead interdisciplinary model of care among Indigenous women in Australia showed no improvements in the rate of low birthweight infants (p 0.855).  (N=121 women)  [Reeve]  Aboriginal Midwifery Access Program (AMAP) determined slightly lower rates of low birthweight babies (18.8% vs 21%) in the intervention group, with no statistical analysis.  (N=130) [Wong]  Mums and Babies program determined no differences in the rates of low birthweight (0.067) in the intervention group,  (N=456)  Panaretto  Similar results obtained in the second cohort (p 0.289)  (N=781) [Panaretto 2007]  The intervention (Birth in Our Community service) significantly reduced the rate of low birthweight (OR 0.60, 95% CI 0.41-0.89, p 0.012).  (N= 766 women)  [Kildea 2021]  The Murri clinic did not influence the rate of low birthweight (p 0.425)  (N=367 women)  [Kildea 2012]  The Strong Women, Strong Babies, Strong Culture Program (SWSBSC) in Australia determined lower rates of low birthweight infants (p 0.014)  (N=829)  [Tursan]  Same results were reported by a different cohort after implementation of the same intervention (low birthweight rate dropped from 19.8% vs 11.3%, p 0.02)  (N=228)  [Mackerras] |  | Hispanic women that had Centering Pregnancy Model (CPM) did not show different birth weights compared to the non-intervention group (p 0.624)  (N=24 women)  [Robertson]  The group ANC in the Somali-born women in Sweden caused a higher rates of SGA to be detected (11% vs 0% p 0.01)  [Ahrne] | Compared to white women, BAME women assigned to two-midwifery care experienced higher rates of low-birth weight infants (6.1% vs 3.2%; p0.002) (N=1620)  [Homer] |
| Newborn wellbeing | The Special Supplemental Nutrition Program reduced the rate of infant death compared to that observed in non-participants (9.6% vs 21%, p <0.001).  (N=5731 women)  [Khanani]  Augmented prenatal care showed no reduction in the rate of neonatal admission to intensive care unit (p 0.11)  (N=318 women)  [Kelman] |  | Mums and Babies program determined no differences in the rates of perinatal death (p 0.864) in the intervention group. (N=456)  Panaretto  The second larger cohort published by the same authors showed significant reduction in the rate of perinatal death (p 0.014)  (N=781)  [Panaretto 2007]  The intervention (Birth in Our Community service) significantly reduced the rate of admission to neonatal nursery (OR 0.69, 95% CI 0.51-0.92, p 0.013); no effect on the rate of neonatal death (p 0.91).  (N= 766 women)  [Kildea 2021]  The Murri clinic reduced the rate of NICU admission among Aboriginals  (p 0.036)  (N=367 women)  [Kildea 2012]  The Aboriginal Maternal and Infant Health Service determined acnon-significant reduction in the perinatal mortality (20.4 to 14.4 per 1000 births)  (N= 689)  [Murphy] |  | Midfetalness practice (active role of women in monitoring quality of fetal movement) did not reduce the rate of Apgar <7 at 5 minutes or NICU admission (aRR 0.22, 95% CI 0.01-1.11; aRR 0.54, 95% CI 0.24-1.08, respectively) (N=169 Somali women) [Askelsson] | Midwife education determined a significant reduction in the rate of arterial pH <7 (aOR 0.27, 95% CI 0.09– 0.81) and a significant increase in the rate of NICU admission (aOR 1.36, 95%CI 1.05–1.76) (N=14.835 women)  [Rasmussen 1]  Non-significant reduction was observed in the rate of neonatal death or other adverse perinatal outcomes with elective IOL at 39 weeks in ethnic minorities (N=47.352 women)  [Muller]  Ethnicity was not predictive of the need for respiratory support within 72 hours after birth in the placebo or study group of the secondary analysis of the RCT about betamethasone in late preterm. (N=500 women)  [Andrikopoulou]  Two-midwives care (Albany Midwifery Practice) for each woman determined a lower perinatal mortality rate in BAME ethnic groups (less than 2.0 per 1000 births vs 8.8-9.8 per 1000 reported in other studies). (N=1620)  [Homer] |
| Breastfeeding |  | General reproductive education was associated with higher rates of breastfeeding with complement (48% vs 31%) (N=69 women)  [McEnery] | The intervention (Birth in Our Community service) did not increase the rate of breastfeeding (p 0.097).  (N= 766 women)  [Kildea 2021]  The Murri clinic did not influence the rate of breastfeeding at discharge (p 0.248)  (N=367 women)  [Kildea 2012] |  |  | Compared to white women, BAME women assigned to two-midwifery care experienced higher rates of mixed or exclusive breast-feeding (90.1% vs 86.8%; p 0.01) (N=1620)  [Homer] |
| OTHER |  |  |  |  |  |  |
| Active engagement with healthcare providers |  | The Asian Mother and Baby Campaign consisting of linkworkers, interpreters and facilitators in the hospitals and community determined no differences in the number of antenatal visits or admissions in pregnancy. Slight improvement in the use of health service in women with a good understanding of English was reported.  (N=213 women)  [Mason] | The intervention (Birth in Our Community service) significantly increased the rate of first visit in the first trimester (OR 1.35, 95% CI 1.05-1.73) and the number of visits (OR 1.49, 95% CI 1.06-2.09, p 0.021).  (N= 766 women)  [Kildea 2021]  The group of Aboriginal that attended the  Murri clinic showed higher numbers of antenatal visits (p 0.007)  (N=367) [Kildea 2012]  The Aboriginal Maternal and Infant Health Service determined an increase in the rate of first visits before 20 weeks (OR 1.2, 95%CI 1.01-1.4, p 0.03)  (N= 689)  [Murphy]  Higher numbers of visits in pregnancy were observed in the group of Aboriginal women that took part in the Mums and Babies program (p <0.001).  (N=781)  [Panaretto 2007] |  | CenteringPregnancy model among Hispanic women determined increased active participation in prenatal care (mean participation score 39.7 vs. 28.1, p < .001), satisfaction with time spent talking with the provider (98% vs. 19%, p < .001), ability to talk to their prenatal care provider in the preferred language (99% vs. 19%, p < .001), acceptability of prenatal care visit waiting time (99% vs. 6%, p < .001), expected prenatal care visit ratio (101.9 vs 83.1, p <0.001).  (N=144)  [Tandon] | Midwife education did not improve engagement of patients with healthcare providers in the forms of “Active management” (adjusted mean difference −0.07, 95 % CI (−0.23-0.09)) or “Navigating the healthcare system” (adjusted mean difference −0.04, 95 % CI −0.20–0.12). Non significant improvement in symptoms awareness was observed. (N=397 women) [Rasmussen 2] |
| Satisfaction with care delivery model | Augmented prenatal care showed significantly higher rates of prenatal care positive rating (p 0.002), higher number of prenatal visits (p 0.001), higher participation in prenatal courses (p <0.001).  (N=318 women)  [Kelman] |  |  |  | Centering Pregnancy Model in Hispanic women did not improve the knowledge of care (p 0.521), the health behaviors (p 0.472) or the self-esteem (p 0.949) prenatally. Post-natally the self-esteem scores were found higher in the non-intervention group (p 0.37).  (N=24 women)  [Robertson]  CenteringPregnancy Model showed increased satisfaction score among Hispanic women (p<0.01)  (N=144) [Tandon] |  |

LDA (low-dose aspirin), PE (preeclampsia), PTB (preterm birth), RCT (randomized controlled trial), SGA (small-for-gestational age), NICU (neonatal intensive care unit)
